# Tissue-specific experimental evolution reveals adaptive trade-offs in the plant vascular pathogen *Clavibacter michiganensis*

**DOI:** 10.1093/ismejo/wrag110

**Published:** 2026-05-07

**Authors:** Raj Kumar Verma, Veronica Roman-Reyna, Nathan Benmoche, Evanson Ngugi, Eduard Belausov, Noa Sela, Maya Bar, Jonathan M Jacobs, Doron Teper

**Affiliations:** Department of Plant Pathology and Weed Research, Institute of Plant Protection, Agricultural Research Organization–Volcani Institute, Rishon LeZion 7505101, Israel; Department of Plant Pathology and Environmental Microbiology, Pennsylvania State University, University Park, PA 16802, United States; Department of Plant Pathology and Weed Research, Institute of Plant Protection, Agricultural Research Organization–Volcani Institute, Rishon LeZion 7505101, Israel; Department of Plant Pathology and Microbiology, The Robert H. Smith Faculty of Agriculture, Food & Environment, The Hebrew University of Jerusalem, Rehovot 7610001, Israel; Department of Plant Pathology and Weed Research, Institute of Plant Protection, Agricultural Research Organization–Volcani Institute, Rishon LeZion 7505101, Israel; Department of Life Sciences, Faculty of Natural Sciences, Ben-Gurion University of the Negev, Beer-Sheva 8410501, Israel; Department of Ornamental Plants and Agricultural Biotechnology, Institute of Plant Sciences, Agricultural Research Organization–Volcani Institute, Rishon LeZion 7505101, Israel; Bioinformatics Unit, Agricultural Research Organization–Volcani Institute, Rishon LeZion 7505101, Israel; Department of Life Sciences, Faculty of Natural Sciences, Ben-Gurion University of the Negev, Beer-Sheva 8410501, Israel; Department of Plant Pathology, The Ohio State University, Columbus, OH 43210, United States; Infectious Diseases Institute, The Ohio State University, Columbus, OH 43210-1358, United States; Department of Plant Pathology and Weed Research, Institute of Plant Protection, Agricultural Research Organization–Volcani Institute, Rishon LeZion 7505101, Israel

**Keywords:** tissue specificity, *Clavibacter*, vascular plant pathogens, experimental evolution, clonal selection, exopolysaccharides, surface-attachment

## Abstract

The plant pathogenic bacterium *Clavibacter michiganensis* (Cm) is a systemic vascular pathogen that colonizes both xylem vessels and the intracellular apoplast during different stages of infection. To identify traits and loci associated with adaptation to these distinct host microenvironments, we conducted tissue-specific experimental evolution. Twenty independent Cm lineages were repeatedly passaged in either tomato stems or leaves to promote adaptation to vascular or apoplastic lifestyles, respectively. After fifteen passages, adapted clones were characterized for virulence and virulence-related traits. These characterizations demonstrated clear differential associations of virulence-associated traits with the adapted tissue. The majority of vascular-adapted clones displayed enhanced surface attachment, reduced cellulase activity, reduced exopolysaccharide (EPS) production, and attenuated virulence on tomato compared to the parent clone. In contrast, apoplast-adapted clones displayed reduced biofilm formation and enhanced EPS production and retained their virulence on tomato. Whole-genome sequencing of all adapted clones revealed candidate loci linked to tissue adaptation. Six of ten vascular-adapted clones carried two independent mutations in *CMM_1284*, a putative HipB/XRE-type transcriptional regulator. A *CMM_1284* marker exchange mutant displayed phenotypes similar to vascular-adapted clones, suggesting a role for this regulator in vascular colonization. Together, these findings highlight the role of phenotypic plasticity in tissue adaptation of plant pathogens, showing that tissue-specific adaptation involves modulation of surface attachment, EPS production, and cell wall–degrading enzymes and suggest a trade-off between vascular persistence, supported by strong surface attachment, and systemic virulence, which depends on bacterial dispersal and migration.

## Introduction

Plant pathogenic microorganisms specialize in colonizing host tissues according to lifestyle: systemic pathogens spread through the vascular system, whereas non-systemic ones remain confined to apoplastic regions. These contrasting lifestyles shape disease etiology, epidemiology, and management. Non-systemic bacteria colonize the apoplast, inducing galls or necrotic lesions [1–3], spread locally by water splash, wounding, or insects, and rarely invade the vasculature [4]. Vascular pathogens specialize in xylem or phloem colonization. Phloem-associated pathogens are insect-transmitted, phloem-restricted, passively transported, and have reduced, low-GC genomes [5]. Xylem-associated pathogens show diverse transmission and tissue specificity: *Xylella* spp. are strictly xylem-limited, whereas vascular Xanthomonads, *Ralstonia* spp., *Erwinia amylovora*, and *Clavibacter* spp. also colonize apoplast tissues [6, 7]. In *E. amylovora*, transitions between xylem and parenchyma are central: it enters the xylem from floral parenchyma, spreads systemically, and exits into bark parenchyma, causing tissue degradation and proliferation [8]. Mechanisms of tissue specificity and lifestyle transitions remain unclear, and even related strains differ in tropism. In *Xanthomonas*, the glycosyl hydrolase gene *cbsA* distinguishes vascular from non-vascular lifestyles [9]. Xylem-associated bacteria produce biofilm-like matrices attaching to vessel walls, essential for systemic spread and implicated in drought-like symptoms by blocking water flow [10]. Mutational analyses show that balancing biofilm formation and dispersal is critical for systemic colonization [11, 12].

Bacterial canker, caused by the Actinomycetota *Clavibacter michiganensis* (Cm), is one of the most destructive tomato diseases [13]. Symptoms include canker lesions on stems and branches, leaf wilting, necrosis, and bird’s-eye spots on fruits [13]. Cm spreads by colonizing xylem vessels, causing vascular collapse through blockage of water-conducting elements [14]. The pathogen relies heavily on secreted hydrolases such as cellulases, xylanases, pectate lyases, and serine proteases, encoded mainly by the *chp/tomA* pathogenicity island (PAI) and plasmids pCM1 and pCM2 [15, 16]. Variation in these regions strongly correlates with pathogenicity [17–19], and disruption of specific hydrolases such as *celA, chpC*, and *pat-1* reduces virulence [20–23]. Microscopy has shown Cm forming biofilm-like aggregates attached to xylem walls [24], suggesting biofilms aid systemic colonization, though their composition, structure, and regulation are poorly understood. Only a few studies indirectly link biofilm formation to regulatory networks such as the stringent response and cell wall integrity [25, 26]. Despite evidence for surface attachment in vitro and *in planta*, adhesion mechanisms remain undefined, and no adhesion-associated macromolecules have been identified. Cm exopolysaccharides (EPS) were hypothesized to contribute to virulence, but this hypothesize has to be tested by functional or genetic analyses [27]. Cm strain NCPPB382 harbours at least four predicted EPS gene clusters [16, 28], but their role in EPS production is not well defined. Cm also colonizes the leaf apoplast, producing blister-like structures [15, 29], though the contribution of this lifestyle to disease progression remains unclear. In this study, we employed a tissue-based experimental evolution approach to identify traits and genetic loci selected for adaptation to either the vascular or apoplastic lifestyle of Cm.

## Materials and methods

### Bacterial strains and plant material

Cm and *E. coli* strains developed and used in this study are listed in Table S1. Cm strains were grown in Luria-Bertani (LB) medium at 28°C with 100 μg/mL trimethoprim [30]. *E. coli* strains were grown in LB medium at 37°C. When required, media were supplemented with 25 μg/mL gentamicin, 75 μg/mL neomycin, 50 μg/mL kanamycin, or 100 μg/mL ampicillin. The plant cultivars used in this study included tomato (*Solanum lycopersicum* cv. Moneymaker), which was used for bacterial adaptation as well as subsequent virulence and competition assays, and eggplant (*Solanum melongena* cv. Black Queen) and *Nicotiana sylvestris*, which were used for hypersensitive response (HR) assays. Plants were grown in a temperature-controlled glasshouse at 25°C under natural light conditions.

### Experimental evolution procedure

Vascular and apoplastic populations were generated through repeated passaging of Cm in tomato stems and leaves by successive cycles of inoculation, isolation, and re-inoculation of naïve plants, using Cm NCPPB382 as the parent strain.

For each vascular passage, bacteria recovered from the previous round were used to inoculate a naïve tomato plant at the four- to six-leaf stage. Inoculation was performed using the “wound inoculation” method by a single stem puncture between the cotyledons using a toothpick pre-soaked in a bacterial suspension of 10^7^ CFU/mL. This approach resulted in an estimated initial bacterial load of 5.55 ± 0.37 log (CFU/g stem) within a 1 mm region surrounding the inoculation site. Plants were then transferred to a growth room, and 14–21 days later, bacteria were re-isolated from 3 mm stem sections located ~6 cm above the inoculation site.

For each apoplastic passage, bacterial suspensions from the previous round were diluted to 10^4^ CFU/mL and infiltrated into the three most recently emerged mature leaves using a needleless syringe. Bacterial cultures were introduced into 4–8 infiltration sites in each leaf (~1 cm^2^ per site). Plants were incubated in a growth room for 7–10 days, after which bacteria were isolated from pooled samples of all infection foci.

For each isolation cycle, bacteria were isolated by homogenization, serial dilution, and plating on LB agar supplemented with 50 μg/mL cycloheximide and 100 μg/mL trimethoprim. Approximately 50–200 newly emerged colonies from each sample were pooled directly from LB agar plates and used for the subsequent passage. Bacteria were collected by scraping colonies into 1 mL MgCl_2_ in a microcentrifuge tube, homogenized by vigorous vortexing, and adjusted to 10^7^ CFU/mL (for vascular adaptation) or 10^4^ CFU/mL (for apoplastic adaptation), and these suspensions was used as the inoculum for the next cycle.

The experiment was carried out using ten parallel bacterial clone lines for each tissue type (vascular and apoplastic), with a total of 15 passages. Each passage was performed in a separate plant (20 plants per passage), and bacterial colonization was quantified at each time point (CFU per gram of stem tissue or per cm^2^ of leaf tissue). Adapted bacterial populations from each passage were stored in 25% glycerol at −80°C. When populations could not be recovered from a given passage due to contamination, bacteria from the previous cycle were retrieved from −80°C stocks, streaked onto LB agar, grown, and the resulting lawn was scraped from the plate and used for inoculation.

For downstream phenotyping and genomic sequencing, bacteria from all parallel lines at passages 5, 10, and 15 were streaked on LB agar, and a single representative colony from each clone was selected. Clones from passage 15 were used for phenotyping, and those from passages 5, 10, and 15 were used for sequencing.

Culture-media adaptation was conducted by pooling bacteria grown in culture media from the previous passage in 1 mL of distilled water, diluting the suspension to OD600 = 0.001, and spreading it on LB agar supplemented with trimethoprim. Plates were incubated for four days and then pooled for the next passage. Media passaging was performed on ten parallel clones for 15 cycles.

### Plant inoculations, disease severity assessments, apoplastic virulence, stem-to-leaf migration and quantification of stem/leaf bacterial populations

The virulence assays were carried out using the wound inoculation method as described previously [31], with minor adjustments. Full description is provided in Supplementary File S1.

Virulence assays were conducted using toothpick-mediated stem wound inoculation and needleless syringe infiltration of tomato leaves. Wilt symptoms in stems were scored at 14 or 21 days post-infiltration (dpi) on a 0–3 scale based on the percentage of affected leaves. Leaf infiltration assays were evaluated at 10 dpi for chlorosis and necrosis on a 0–3 scale, and representative leaves were photographed. Bacterial populations were quantified from stem segments or leaf disks by plating serial dilutions, standardized to tissue weight or surface area. HR assays were performed in eggplant and *N. sylvestris* by syringe infiltration, with symptoms recorded at 36 h.

### Cloning and bacterial manipulation

All plasmids and oligonucleotides produced and used in this study are listed in Tables S2 and S3, respectively. Detailed information regarding plasmid construction is provided in Supplementary File S1. The plasmid pMA-RQ:Cmp [18] served as the backbone for constructing marker exchange, overexpression, reporter plasmids, and antibiotic labelling for competition assays. The marker exchange construct pCMAT:1284 was generated to obtain a *CMM_1284* marker exchange mutant, which was confirmed by PCR. For overexpression, the backbone was modified with a promoter and a genomic integration site, producing derivatives such as pCMIARG, including a CelA-3 × HA fusion confirmed by western blot. Reporter plasmids carrying *celA* and *gyrB* promoters fused to the *uidA* (GUS) gene were built in the Cm–*E. coli* shuttle vector pHN216 [32] and used for promoter activity assays. All constructs were introduced into Cm by electroporation, as described previously [31].

### *In planta* bacterial competition assays

Bacterial competition assays were conducted *in planta*, comparing Cm WT with representative vascular-adapted (S2, S4, S9) and apoplast-adapted (L2, L7, L9) clones. Cm WT was introduced with pCMIAR, whereas clones S2, S4, S9, L2, L7, and L9 were introduced with pCMNTR, conferring gentamicin and neomycin resistance, respectively. For competition assays, gentamicin-resistant Cm WT was mixed 1:1 with each neomycin-resistant adapted clone, CmΩ1284, or gentamicin-resistant Cm WT alone (control) at final concentrations of 5 × 10^7^ CFU/mL (vascular virulence) or 10^4^ CFU/mL (apoplastic virulence), and plants were inoculated as described above. Cm WT/adapted clone ratios were determined by spotting isolated bacteria on triplicate LBA plates containing trimethoprim, trimethoprim + gentamicin, or trimethoprim + neomycin to quantify total populations and the fraction of gentamicin-resistant colonies (Cm WT). Ratios were measured at time 0 in inoculum cultures and at 14 dpi in stems at the point of inoculation (POI) and 5 cm above the POI for vascular virulence, and at 0 and 7 dpi in leaf tissues at the infiltration site for apoplastic virulence.

### EPS quantification, surface attachment, and plate halo assays

Detailed descriptions of experimental procedures used for in vitro characterization are provided in Supplementary File S1. Briefly, EPS quantification was conducted on Cm bacteria grown in 5% sucrose-supplanted LB plates, using the phenol-sulfuric acid estimation method [33]. Surface attachment/biofilm assays were performed using 24 well plates supplemented with Cm cultures previously grown on LB broth. Attachment was assessed and quantified after static incubation using the crystal violet staining method as previously described [34] with modifications. Exoenzyme activity of Cm clones was tested by plate halo assays on media containing specific substrates for cellulase, amylase, xylanase, polygalacturonase, protease, or lipase, with halos visualized by staining and quantified by diameter measurements [35]. Siderophore production was assessed using the CAS agar assay [36] under iron-limiting conditions, with activity visualized as yellow/orange halos.

### *In situ* localization of *C. michiganensis* during infection using confocal laser scanning microscopy

Cm clones NCPPB382, S2, and S4 were transformed with the *E. coli*–*Clavibacter* shuttle vector pK2–22 [24], expressing EGFP under the control of the p*CMP1* promoter. *In situ* localization of bacteria was monitored in infected plants using an Olympus IX81 confocal laser scanning microscope equipped with a GFP filter set. To visualize bacterial localization in stem tissues, ~0.1 mm-thick horizontal and vertical cross-sections were collected approximately 3 cm above the inoculation site. For leaf tissues, ~0.5 cm^2^ sections were excised from areas exhibiting healthy, wilted, or necrotic phenotypes and directly mounted on microscope slides for imaging.

### Promoter activity assays and gene expression analysis

For promoter activity and gene expression assays, Cm WT, S2, S4, and S9 strains carrying pHN:p*celA*:*GUS* or pHN:*pgyrB*:*GUS*, were grown overnight in LB, washed twice with distilled water, resuspended in M9 medium to induce virulence-associated gene expression [37], and incubated at 28°C with rotation for 24 h.

For promoter activity, OD600 was measured, and 1.5 mL of each culture was lysed with 1 mg/mL lysozyme followed by sonication (SONIC-150 W, MRC). Lysates were centrifuged, and 80 μl of supernatant was mixed with 10 μl 10× Tris-based saline (pH 7.4) and 10 μl 10 mM PNPG. Reactions were incubated at 37°C and monitored for yellow pigmentation, then stopped with 50 μl 1 M sodium bicarbonate. Absorbance at 405 nm was measured, and activity was calculated as OD405/(time × OD600) and standardized to WT activity.

For gene expression, total RNA was extracted from culture supernatants using the Hybrid-R Kit (GeneAll), reverse transcribed with the UltraScript cDNA Synthesis Kit (PCR Biosystems), and amplified using Fast SYBR qPCR Master Mix (Biogate) on a QuantStudio 3 system (Applied Biosystems) with gene-specific primers (Table S3). Expression was normalized to *gyrA* and calculated with the comparative Ct method [38].

### Genome sequencing, assembly, and comparative genomic analysis

Three independent Cm NCPPB382 (WT) clones and tissue-adapted singular clones collected after 5, 10, and 15 passages were subjected to whole-genome sequencing. DNA was extracted from 10 mL overnight LB cultures using the Wizard Genomic DNA Purification Kit (Promega). Libraries were sequenced on NextSeq 2000 System (Illumina) with 150 bp paired-end at the Applied Microbiology Services Laboratory (Ohio State University). Reads were cleaned with Trimmomatic [39] and assembled using Unicycler v0.5.0 and SPAdes v3.15.5 [40, 41]; contigs <200 bp were removed. Genome completeness was assessed with BUSCO v5 (micrococcales_odb10) [42]. Genomes were deposited in the NCBI BioProject database under the ID PRJNA1333927. Genomic alterations were identified with Snippy (https://github.com/tseemann/snippy) and confirmed by BWA [43] against the NCPPB382 reference (GCA_000063485). Passage 10 of clone S6 and passage 5 of clone S10 were excluded due to poor sequencing quality. All INDELs and SNPs are listed in Table S4, excluding mutations found in any of the three resequenced WT clones. Ten representative ORF mutations were confirmed by Sanger sequencing (Table S4, ^*^). The corresponding regions were PCR-amplified from Cm WT and adapted clones using specific primers (Table S3), sequenced (Hylabs Laboratories), and compared with assembled genomes.

### Amplicon sequencing

Glycerol stocks of populations collected at cycle 15 from S2, S4, S9, L2, L3, L10, and Cm WT were used as templates for PCR amplification (25 cycles) of 200–300 bp regions surrounding SNPs in ***CMM_1284, CMM_2466, CMM_1971, CMM_0376*, and *CMM_0813*** using gene-specific primers (Table S3) fused to SP1/2 linkers. Amplicons from the five genes were pooled per population and submitted to Syntezza Bioscience (Jerusalem, Israel) for enrichment PCR to add sequencing adapters and dual-index barcodes. Libraries were converted to single-stranded circular (ssCir) DNA using the Universal Library Conversion Kit (App-A, MGI Tech, Shenzhen, China) and sequenced on the MGI-G400 platform using the G400 App-D FCL PE150 kit (MGI Tech) in paired-end mode (2 × 150 bp), generating ~1 million reads per sample. For analysis, forward and reverse primer sequences were removed using Cutadapt [44], allowing 10% mismatches, and reads lacking primer sequences were discarded. Paired-end reads were then merged using fastp [45] with the parameters—overlap_len_require 20 and—overlap_diff_limit 5. Processed reads were aligned to a five-gene reference using BWA-MEM [46]. Resulting BAM files were sorted and indexed using SAMtools v1.16.1 [47]. Base counts were extracted using bam-readcount [48]. Full sequencing data is available in Table S5.

### Data processing, visualization, and statistical analyses

All data were obtained from 2–4 independent experiments, each comprising at least three biological replicates; specific details on sample size and number of repeats are provided in the figure legends. Statistical analyses and data visualization were performed using pooled data from all biological replicates across all experimental repeats. Continuous data, including plant bacterial populations, plate halo assays, gene expression analysis, promoter activity assays, and relative populations in competition assays, are presented as box plots in which lower and upper quartiles define the box boundaries, with central lines and “o” symbols indicating medians and individual data points, or alternatively as bar graphs showing means ± standard error (SE). Bacterial plant populations were log10-transformed and RT-qPCR expression data were log2-transformed, and both were analyzed and presented following logarithmic transformation. All continuous data were analyzed using either a two-tailed Mann–Whitney U test or a two-tailed Welch’s *t-*test, as specified in the figure legends. Ordinal and binary data, including disease scores and bacterial presence/absence assays, are presented as stacked bar graphs indicating relative proportions (%), and were analysed using a chi-square test comparing the distribution of all score categories. Statistical significance was defined as *P* < .05, comparing adapted clones to the Cm WT parent or, in in planta bacterial competition assays, comparing time points at 7 or 14 dpi to time 0. During data presentation, statistical significance is indicated by asterisks corresponding to *P* value thresholds (^*^P value <0.05, ^**^P value <.01, ^***^P value <.001). Graphs were generated using Microsoft Excel, and statistical analyses were performed using Prism (GraphPad) and/or Microsoft Excel.

## Results

### Cm migrates from the xylem to apoplast tissue during infection

Cm is traditionally classified as a xylem-inhabiting bacterium [7]. However, in contrast to xylem-restricted bacteria such as *Xylella fastidiosa*, Cm has also been reported to colonize intracellular apoplastic spaces within host tissues [7, 24, 49]. To assess Cm’s tissue localization, GFP-labelled Cm [24] was monitored for colonization throughout the plant following infection.

**Figure 1:**
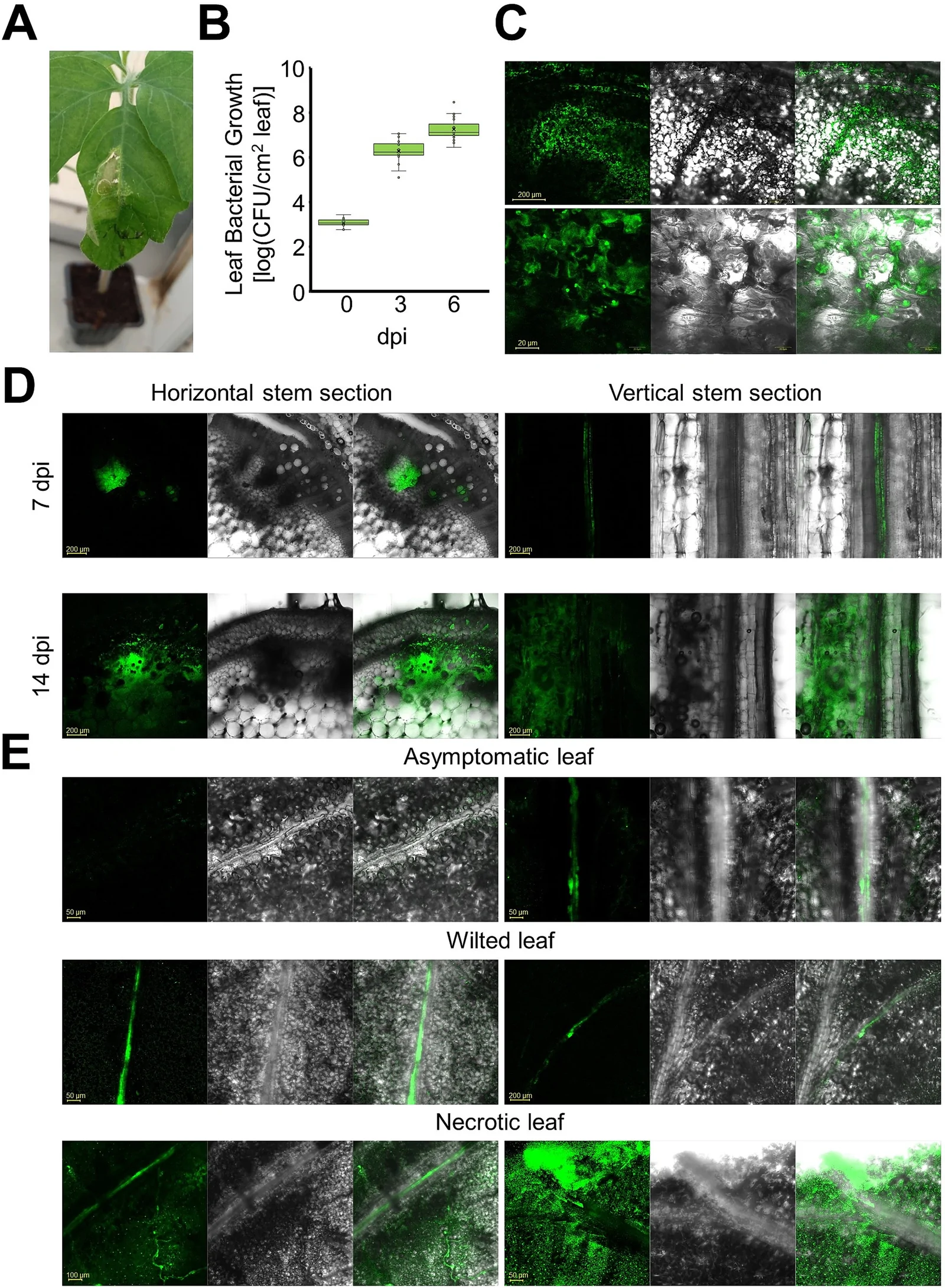
*In situ* localization of Cm bacteria during infection. (A–C). Cultures (10^4^ CFU/mL) of GFP-labelled Cm were infiltrated into six-leaf-stage tomato leaves using a needleless syringe. (A) Representative leaf photographed 10 days post-infiltration (dpi). (B) Bacterial population at the infiltration site on days 0, 3, and 6 dpi. Data represent 26 biological repeats pooled from three independent experiments. (C) Infiltrated leaf tissues were visualized by confocal laser scanning microscopy using a GFP filter at 7 dpi. (D, E) stem areas between the cotyledons of four-leaf-stage tomato plants were wound-inoculated using toothpicks soaked in 10^7^ CFU/mL GFP-labelled Cm bacterial cultures. Bacteria were visualized by confocal laser scanning microscopy with a GFP filter. (D) *In situ* localization of Cm at 3 cm above the infection site, visualized in horizontal and vertical stem cross sections of Cm-inoculated plants at 7 dpi (prior to symptom development) and 14 dpi (when symptoms are apparent). (E) *In situ* localization of Cm in asymptomatic, wilted, and necrotic leaves at 14 dpi. The right and left panels show the two most common localization patterns at leaves during the described symptomatic stages. All depicted data represent at least 10 biological repeats conducted in at least two (C) or three (A, B, D, E) independent experiments.

We examined Cm colonization of the apoplast by syringe-infiltrating bacteria into tomato leaves (Fig. 1A–C). Cm caused localized water-soaked lesions that became necrotic within 5–10 days (Fig. 1A), accompanied by a ~ 10 000-fold population increase by 3 dpi (Fig. 1B). Confocal microscopy showed Cm cells in intracellular apoplastic spaces of leaf parenchyma (Fig. 1C), confirming its ability to thrive during apoplastic infection. Like many apoplastic pathogens, Cm-induced lesions stayed confined to infiltrated areas, and inoculated plants showed no wilt or canker symptoms typical of systemic infection. Thus, whereas Cm can act systemically, direct apoplastic introduction rarely leads to systemic spread.

We examined Cm localization during systemic infection by wound-inoculating stems with GFP-labelled Cm between the cotyledons. *In situ* monitoring was done 3 cm above the infection site at 7 dpi, before severe symptoms, and at 14 dpi, when ~75% of leaflets were wilted and stem cankers were apparent. At 7 dpi, Cm was mainly in xylem vessels with little or no presence in pith parenchyma, consistent with its xylem-restricted lifestyle (Fig. 1D, upper panels). By 14 dpi, bacteria had burst from the xylem into pith parenchyma intracellular spaces (Fig. 1D, lower panels). In distal leaf tissues at 14 dpi, asymptomatic leaflets showed no bacteria or restriction to the main vein, wilted leaflets contained bacteria in vascular bundles or nearby tissues, and necrotic leaflets were heavily colonized in both vascular bundles and apoplastic parenchyma (Fig. 1E). Together, these findings indicate that Cm exits the vascular system into apoplastic parenchyma during late infection, and that xylem escape is key to its infection cycle.

### Experimental evolution of Cm under distinct vascular and apoplastic niches

**Figure 2:**
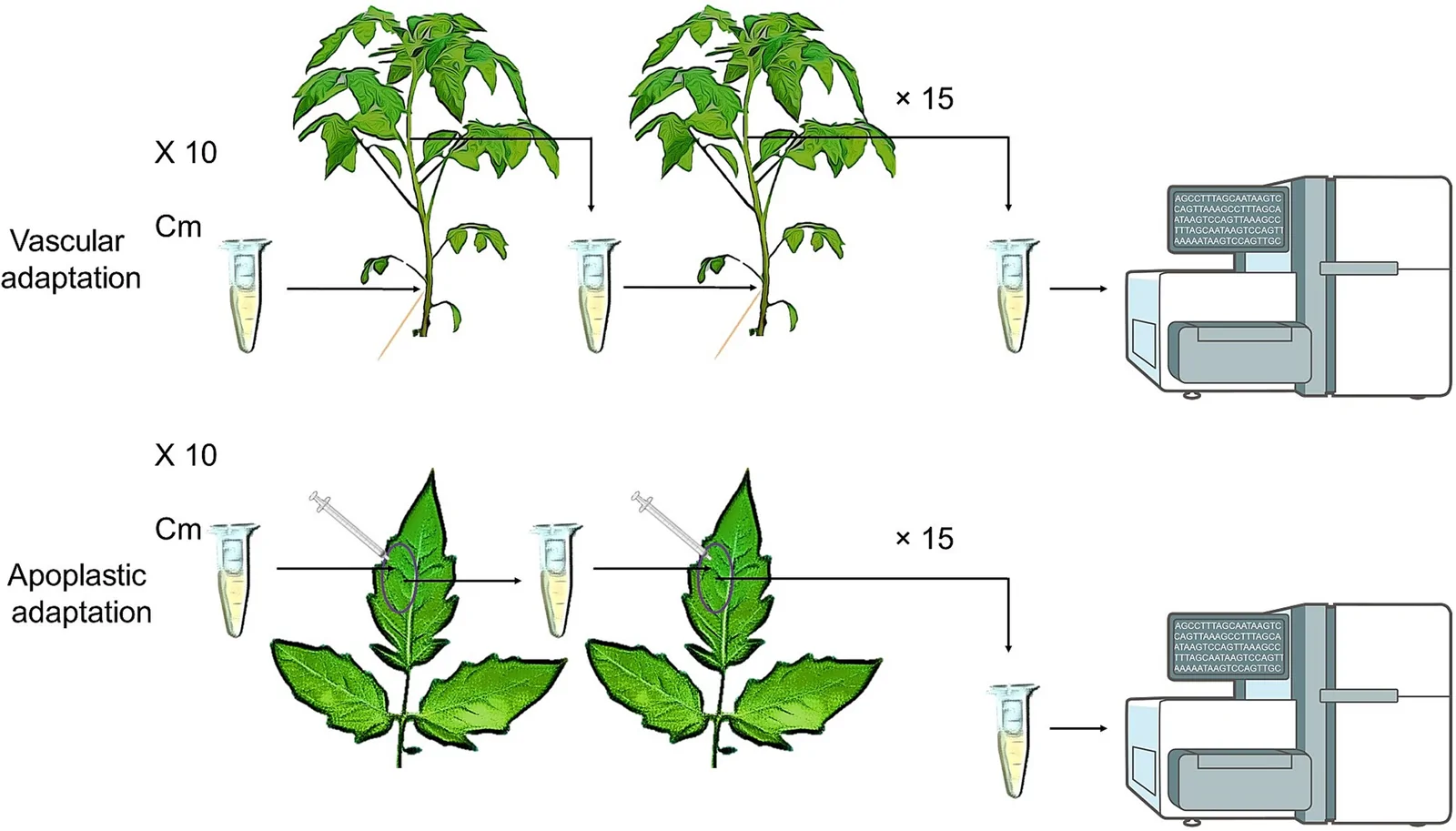
Tissue-specific experimental evolution design. Cartoon illustrating the experimental evolution procedure used in the study. Ten parallel Cm lines underwent repeated inoculation and re-isolation passages lasting 14–21 days, performed via stem (upper panel, vascular adaptation) and leaf (lower panel, apoplastic adaptation) inoculations. The cartoon was created with the assistance of cartoon photo editor (https://play.google.com/store/apps/details?id=com.gamebrain.cartoon&hl=en) and NIAID Visual & Medical Arts (bioart.niaid.nih.gov/bioart/386, bioart.niaid.nih.gov/bioart/506).

The xylem and apoplast microenvironments within a plant host differ greatly and pose unique challenges to invading pathogens [6]. Because Cm can thrive and transition between these habitats, it likely possesses traits supporting survival in each, along with phenotypic plasticity enabling the shift. We hypothesized that traits or loci associated with each environment could be identified by experimentally evolving Cm under repeated exposure to vascular or apoplastic conditions. To test this, we generated 20 parallel tissue-adapted Cm populations by repeated passaging in vascular or apoplastic niches. Ten clones were evolved in vascular tissue (S1–S10) and ten in the leaf apoplast (L1–L10). Experiments used Cm NCPPB382, hereafter referred to as Cm WT or parent clone. Vascular-adapted clone populations were passaged through distal stems: tomato stems were inoculated via toothpick puncture, and bacteria were re-isolated 14–21 days later from tissues 6 cm above the inoculation site. Isolates were pooled and used to inoculate new hosts for the next passage (Fig. 2). Apoplast-adapted clone populations were passaged by infiltrating diluted Cm suspensions (10^4^ CFU/mL) into leaf apoplast with a needleless syringe. Bacteria were re-isolated 7–10 days later from the infiltration area, pooled, and used to inoculate new hosts (Fig. 2). Parallel passaging continued for 15 cycles, after which one representative clone from each lineage was selected for phenotypic and genomic analyses.

### Vascular-adapted clones exhibit reduced vascular and apoplastic virulence

**Figure 3:**
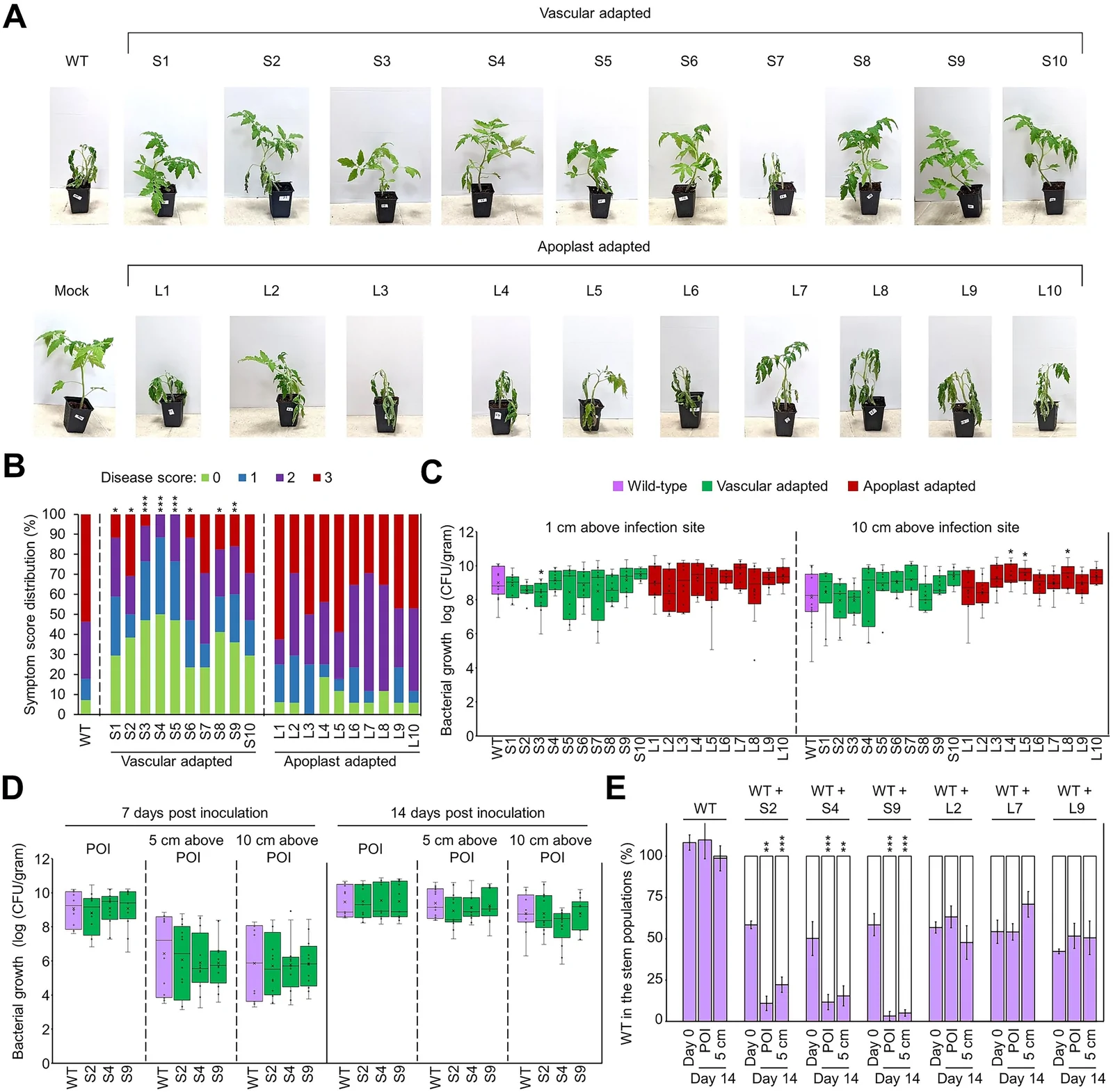
Vascular adaptations affect vascular virulence. (A–D) four-leaf stage tomato plants were wound-inoculated with the indicated vascular-adapted clones (S1-S10), apoplast-adapted clones (L1-L10), and Cm 382 (WT) at the stem areas between the cotyledons. (A) Representative plants (out of 14 repeats) were photographed at 21 dpi. (B) Wilt symptoms were scored by the percentage of wilted leaves according to the following scale: 0–no wilting, 1 = 1%–25%, 2 = 25%–50%, 3 = 51%–100%. The graph depicts the distribution of 14 repeats for each clone pooled from two experiments. “^*^” represent significant difference (chi-squared test, ^*^P value <.05, ^**^P value <.01, ^***^P value <.001) compared to WT. (C) Bacterial growth in stems at 1 and 10 cm above the infection sites at 21 dpi. Box plots represent the 25%–75% interquartile range, and lines indicate the median of 10 replicates pooled from two experiments. “^*^” indicates significant differences compared to WT at the same sampling site (U test, *P* value <.05). (D) Bacterial growth of the indicated clones in stems at the POI, 5 cm above the POI, and 10 cm above the POI was monitored at 7 and 14 dpi. Data represent 10 replicates pooled from two experiments. No significant differences were observed compared to Cm WT at the corresponding collection points (U test, *P* value <.05). (E) Tomato plants were co-inoculated at the stem areas between the cotyledons with a 1:1 mixture of WT and the indicated adapted clones, or with WT alone. The graph shows the percentage of WT within Cm populations in the inoculum (time 0) and in stems at the POI and 5 cm above the POI at 14 dpi. Bar graphs represent mean ± SE of at least 10 replicates pooled from three experiments. “^*^” indicates significant differences compared to day 0 (paired U test, ^*^P value <.05, ^**^P value <.01, ^***^P value <.001).

We investigated whether adaptation to specific plant tissues affected virulence and colonization in vascular and apoplastic tissues. After 15 passages, representative adapted clones from each of the clone population linages and the parent Cm WT were inoculated into tomato stems or leaves, and plants were monitored for symptoms and bacterial growth. For vascular virulence, stems were toothpick-inoculated and wilt symptoms were monitored for 21 days. Bacterial titers were also measured 1 and 10 cm above the inoculation site. Whereas apoplast-adapted clones (L1–L10) caused wilt symptoms comparable to WT, eight vascular-adapted clones (S1, S2, S3, S4, S5, S6, S8, S9) caused reduced wilt symptoms (Fig. 3A and B), despite unchanged bacterial titers at 21 dpi (Fig. 3C). To test kinetic systemic spread, bacterial loads at the POI and 5 and 10 cm above the POI were measured at 7 and 14 dpi in three virulence-attenuated vascular-adapted clones (S2, S4, S9). Colonization of the three clones matched Cm WT at the three collection points at 7 and 14 dpi, indicating that vascular adaptation did not significantly affect stem systemic spread (Fig. 3D). For apoplast virulence, bacteria were syringe-infiltrated into leaves, and symptoms were scored for 10 days. Symptoms caused by apoplast-adapted clones resembled those of the WT (Fig. S1A and B). Four vascular-adapted clones (S2, S3, S4, S5) caused weaker or delayed symptoms, whereas S7 caused stronger, earlier lesions (Fig. S1A and B). However, all clones eventually reached similar apoplastic titers (Fig. S1C). To further assess whether tissue-adapted clones harbour a competitive advantage over the parent clone, we conducted bacterial competition assays *in planta* using representative vascular (S2, S4, S9) and apoplast (L2, L7, L9) adapted clones. Plants were co-infected with Cm WT at a 1:1 ratio, and relative populations were monitored at 14 and 7 dpi for vascular and apoplast inoculations, respectively. In vascular colonization, all three vascular-adapted clones showed a local (POI) and systemic (5 cm above POI) advantage and were overrepresented in stem populations compared to Cm WT, whereas the relative ratios of apoplast-adapted clones remained similar to Cm WT (Fig. 3E and S2). In contrast, all clones showed similar ratios to Cm WT in leaf apoplast growth, except S9, which outcompeted Cm WT (Figs. S1D and S2). Overall, vascular adaptation produced clones with attenuated virulence in both tissues, whereas apoplast adaptation preserved full virulence.

### Vascular-adapted clones demonstrate reduced stem-to-leaf migration

**Figure 4:**
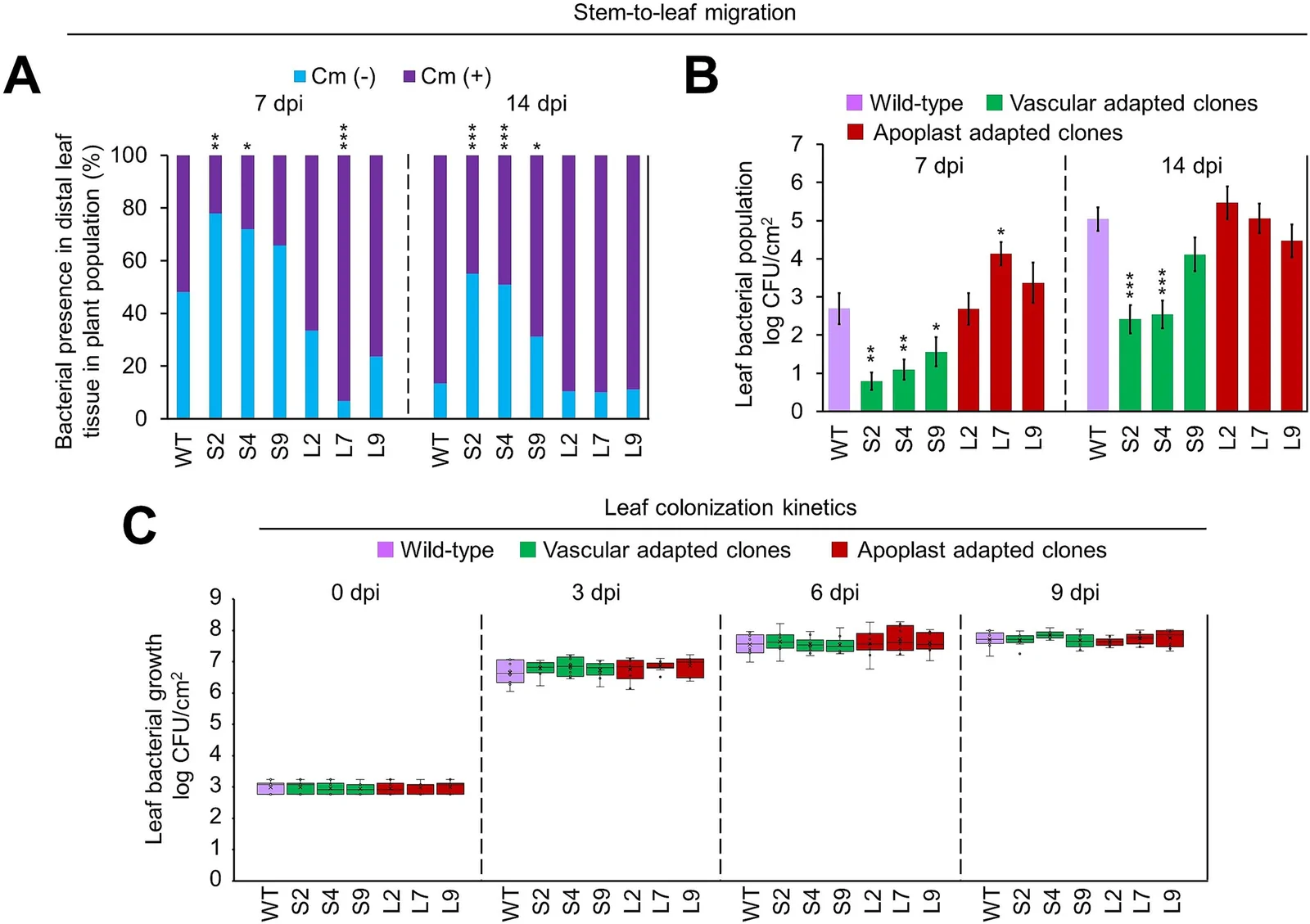
Vascular and apoplastic adaptations affect stem-to-leaf migration. (A, B) four-leaf stage tomato plants were wound-inoculated with the indicated vascular-adapted clones (S2, S4, and S9), apoplast-adapted clones (L2, L7, and L9), and Cm382 (WT) at the stem areas between the cotyledons. Bacteria were isolated and quantified from pooled samples of terminal leaflets from the second, third, and fourth true leaves of each plant at 7 and 14 days post inoculation (dpi). Data represents at least 30 repeats pooled from five (for WT, S2, S4, S9) or three (for L2, L7, and L9) experimental repeats. (A) Stacked bar graphs represent presence/absence of bacteria in the sampled leaflets at 7 and 14 dpi. “^*^” indicates that data were significantly different compared to Cm WT (chi-squared test, ^*^P value <.05, ^**^P value <.01, ^***^P value <.001) at the same dpi. (B) Bar graphs represents the averages and standard errors of leaf bacterial populations. “^*^” indicates that bacterial populations were significantly different compared to Cm WT (U-test, ^*^P value <.05, ^**^P value <.01, ^***^P value <.001) at the same dpi. (C) Six-leaf stage tomato leaves were inoculated with the indicated clones through infiltration of bacterial cultures (10^4^ CFU/mL) using a needleless syringe. Bacterial growth in the infiltration sites at 0, 3, 6, and 9 dpi. Data represents 10 repeats for each clone pooled from two experiments. No significant differences (U-test) were observed between each of the clones to the WT.

Most vascular-adapted clones colonized and migrated systemically through the stem similarly to the WT parent, suggesting that their reduced wilting symptoms are not solely due to limited stem colonization. Because our *in situ* localization experiments linked wilting to bacterial presence in leaves (Fig. 1), we hypothesized that their attenuated virulence may reflect reduced migration from stem to leaves. To test this, tomato plants were stem-inoculated with Cm WT, vascular-adapted clones (S2, S4, S9), and apoplast-adapted clones (L2, L7, L9). Bacterial presence and populations were monitored in pooled terminal leaflets from the second to fourth true leaves at 7 and 14 dpi. Vascular-adapted clones showed lower frequencies and titers compared to WT, whereas apoplast-adapted clones colonized leaves at equal or higher levels (Fig. 4A and B). At 7 dpi, over 50% of leaflets were colonized by WT and apoplast-adapted clones but only 20–30% by vascular-adapted clones. By 14 dpi, colonization in WT and apoplast-adapted clones exceeded 90%, whereas S2 and S4 reached ~50% and S9 ~ 75% (Fig. 4A). These results suggest vascular-adapted clones either migrate more slowly from stems or struggle to establish in leaves. To distinguish these, we directly infiltrated leaves with all seven clones and tracked colonization at 3, 6, and 9 dpi. No significant differences were detected (Fig. 4C), indicating reduced leaf colonization results from impaired stem-to-leaf migration rather than defective leaf colonization.

We examined if this defect reflected altered tissue localization. GFP-labelled S2 and S4 were compared with WT in distal stem and leaf tissues (Fig. S3). No clear differences in localization were observed, and patterns correlated with leaflet symptoms. Consistent with isolation data, bacteria were absent from many asymptomatic leaflets inoculated with S2 and S4, though this also occurred at lower frequency in the WT. In summary, vascular-adapted clones show a pronounced defect in stem-to-leaf migration, likely underlying their attenuated virulence.

### Tissue-adapted clones demonstrate altered colony morphology, production of exopolysaccharides, and surface attachment

Considering that vascular adaptation altered both vascular and non-vascular virulence, we hypothesized that traits linked to pathogenicity and xylem association were also modified. We therefore examined EPS production, surface attachment, exoenzyme activity, siderophore production, HR elicitation in non-hosts, and expression of virulence-associated genes. Siderophore production and HR induction were unchanged in adapted clones relative to Cm WT (Fig. S4), but several traits shifted in a tissue-dependent manner.

**Figure 5:**
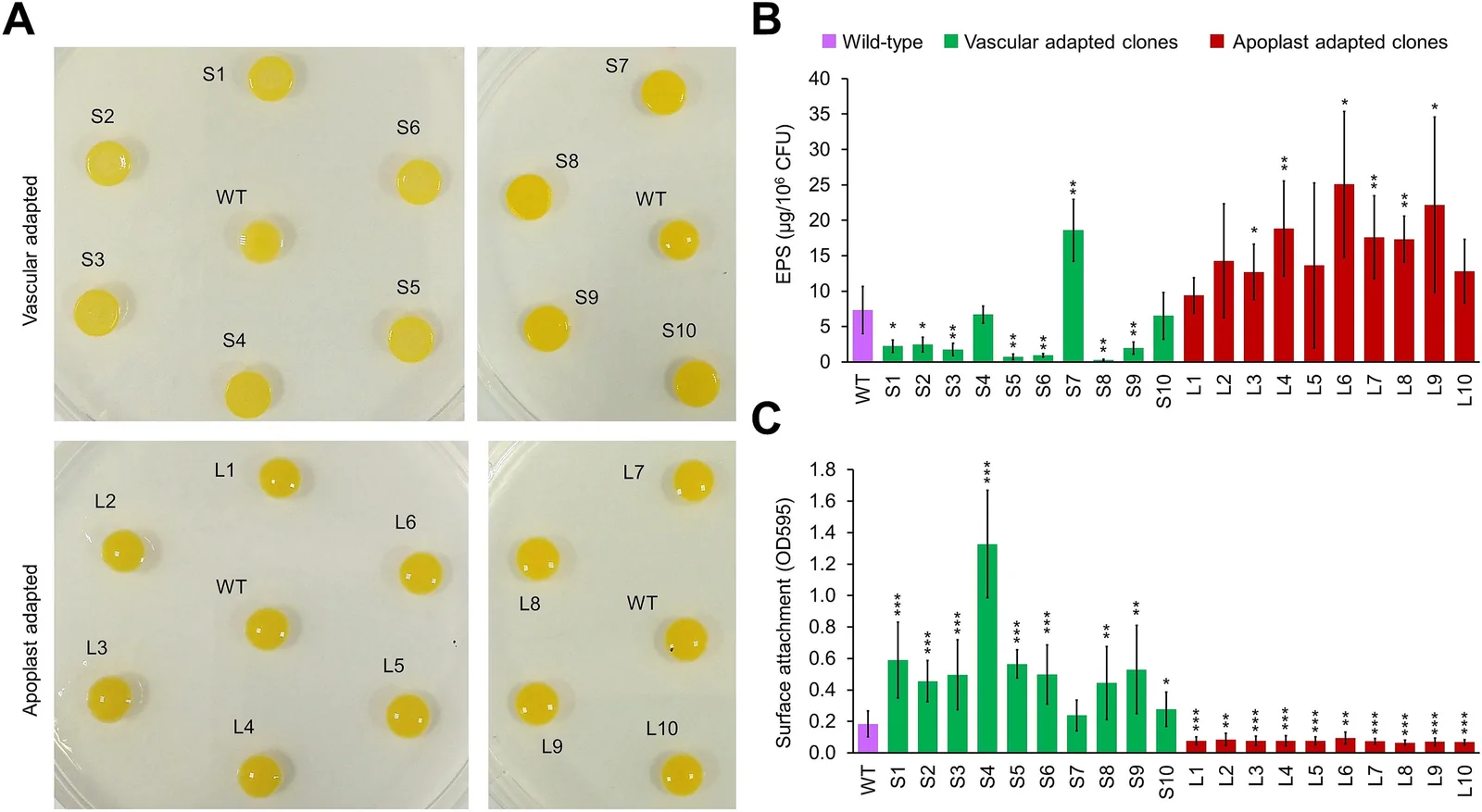
Vascular and apoplastic adaptations effect on EPS production and surface attachment. (A, B) Cm WT and the indicated vascular- and apoplast-adapted clones were spotted (OD600 = 1) on LB agar supplemented with 5% sucrose and incubated for four days. Plates were photographed at 5 dpi (A) and EPS was quantified in the resulting clones (B) using phenol–sulfuric acid hydrolysis assays following by colorimetric quantification and standardization to bacteria count. (C) Bacteria were grown on LB media, standardized to OD600 = 0.5 and incubated in 24 well plates without shaking for 10 days. Supernatants were removed, wells were washed three times with distilled water, and attached bacteria were stained with crystal violet to quantify surface attachment followed by colorimetric quantification. (B, C). Graphs represent one out of three experimental repeats each composed of three (B) or four (C) biological repeats showcasing similar results. “^*^” represent significant difference (U-test, ^*^P value <.05, ^**^P value <.01, ^***^P value <.001) compared to Cm WT.

On rich or minimal media, eight of ten vascular-adapted clones (S1, S2, S3, S4, S5, S6, S8, S9) that also showed attenuated vascular virulence formed dry, condensed colonies, whereas apoplast-adapted clones resembled WT with wet, glossy colonies (Fig. 5A). This suggested reduced EPS secretion or altered EPS composition. Quantification confirmed that seven vascular-adapted clones produced less EPS (Fig. 5B), whereas six apoplast-adapted clones produced significantly more. To further investigate this pattern, we examined the transcriptional expression of genes predicted to be involved in EPS production [28] in representative vascular-adapted clones (S2, S4, and S9) with low EPS yields and observed significant reductions in specific target genes in some clones, but not others (Fig. S5).

Because EPS production is traditionally associated with surface attachment and biofilm formation [50, 51], we conducted in vitro surface attachment assays, which also serve as a proxy for biofilm formation, on all adapted clones using a 24-well crystal violet staining assay (Fig. 5C). Unexpectedly, vascular-adapted clones with dry colonies and low EPS showed stronger attachment than WT, whereas apoplast-adapted clones, which produced more EPS, had weaker attachment (Fig. 5C). These results suggest that, unlike in other systems, EPS may reduce attachment and biofilm formation in Cm. Overall, EPS production and surface attachment appear shaped by tissue-specific selective pressures: vascular adaptation favoured high attachment and low EPS, whereas apoplast adaptation favoured low attachment and high EPS.

### Vascular-adapted clones demonstrate reduced cellulase and amylase activity

Cm bacteria heavily depend on secreted hydrolases such as CAZymes and serine proteases to cause disease [15]. Therefore, we monitored plate exoenzyme activity by halo assays on multiple substrates and transcriptional expression of predicted virulence associated serine proteases and CAZymes.

**Figure 6:**
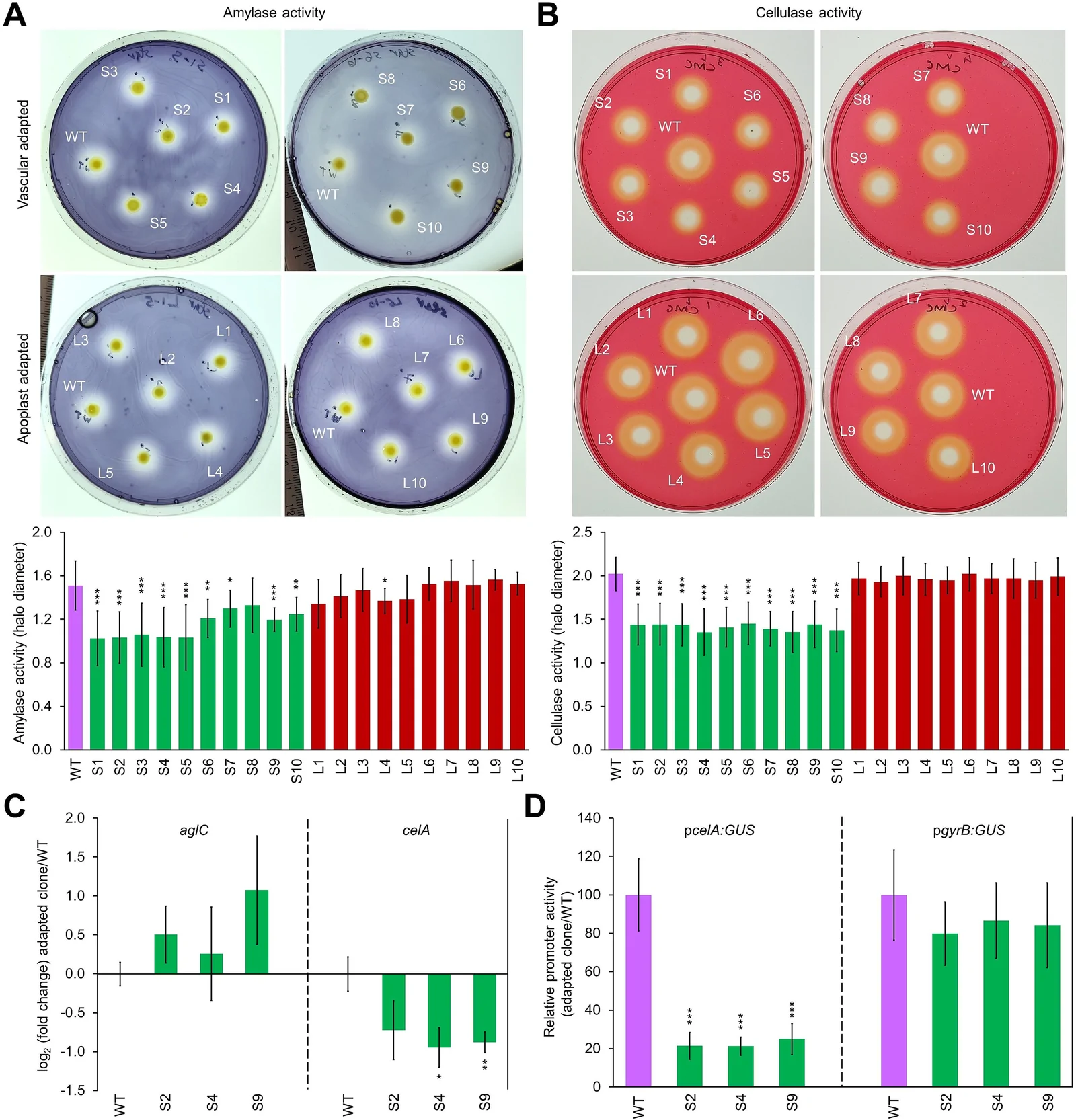
Vascular-adapted clones demonstrate reduced amylase and cellulase activity. (A, B) *Cm* WT and the indicated vascular- and apoplast-adapted clones were spotted (OD600 = 1) onto M9 medium supplemented with 0.1% starch (A) or carboxymethyl cellulose (CMC) (B), and 0.01% sucrose, then incubated for seven (A) or four (B) days. Plates were stained with 0.1% iodine (A) or Congo red (B), photographed (upper panels), and halo diameters were measured with a ruler to assess starch or CMC degradation (lower panels). Graphs represent means ± SE of at least nine replicates pooled from two independent experiments. (C) mRNA transcript abundance of the putative amylase-encoding gene *aglC* (CMM_2797) and the cellulase-encoding gene *celA* (pCM1_0020) was quantified by RT-qPCR in the indicated *Cm* cultures after 24 h incubation in sucrose-supplemented M9 medium. *gyrA* (CMM_0007) mRNA levels were used for normalization. Graphs represent means ± SE of relative transcript abundance compared to *Cm* WT, based on six independent biological replicates pooled from two independent experiments. (D) GUS activity was measured in the indicated *Cm* cultures harboring plasmids carrying *gyrB* (CMM_0006) or *celA* promoter-GUS fusions after 24 h incubation in sucrose-supplemented M9 medium. Graphs represent means ± SE of relative GUS activity compared to *Cm* WT, based on nine independent biological replicates pooled from three independent experiments. (A–D) “^*^” indicate statistically significant differences compared to *Cm* WT (U-test for A, B, and D, Welch’s *t-*test for C, ^*^P value <.05, ^**^P value <.01, ^***^P value <.001).

Halo assays revealed no tissue-adaptation bias in lipase and xylanase activity in the adapted clones (data not shown). However, a clear association was observed between tissue adaptation and amylase and cellulase activity, visualized using starch and CMC degradation, respectively (Fig. 6A and B). Specifically, all vascular-adapted clones demonstrated reduced amylase and cellulase activity, whereas, with the exception of L4 amylase activity, no significant difference was observed in the apoplast-adapted clones compared to the WT (Fig. 6A and B). Although amylase activity has yet to be subjected to any study in Cm, previous studies reported that cellulase activity in Cm is mediated by the secreted endo-beta-1,4-glucanase CelA, and disruption of *celA* abolishes cellulase activity in CMC halo assays, leading to a significant reduction in virulence with minimal impact on *in planta* growth [20, 52]. Therefore, we monitored the transcriptional expression of *celA* and the putative amylase-encoding gene *aglC* in the representative vascular-adapted clones S2, S4, and S9 by RT-qPCR and found that *celA* expression was significantly reduced in S4 (*P* value = 0.0191) and S9 (*P* value = .0087), but not in S2 (*P* value = .137), whereas *aglC* expression was not significantly affected in S2 (*P* value = .239), S4 (*P* value = 0.698), or S9 (*P* value = .198) (Fig. 6C). To further confirm that *celA* expression is reduced in the vascular-adapted clones, Cm WT, S2, S4, and S9 were introduced with a plasmid carrying transcriptional fusion of the *celA* or the *gyrB* (used as a control) promoter to a *uidA* (*GUS*) reporter gene and monitored for GUS activity. Supporting transcriptional data, *celA* promoter activity was significantly reduced in the vascular-adapted clones compared to Cm WT whereas the *gyrB* promoter activity was not significantly altered (Fig. 6D). Next, we tested the transcriptional expression of additional CAzyme coding genes *xysA* and *nagA*, which did not change significantly compared to the Cm WT and three Chp/Pat-1 family serine proteases: *chpC* and *chpE* that were reduced in S2 and S4, and *chpG* that was unaltered (Fig. S5). These findings suggest that vascular adaptation resulted in reduced exoenzyme activity and expression of specific secreted virulence factors.

### Overexpression of *celA* in vascular-adapted clones only partially restores virulence

The cellulase CelA has been previously established as a key virulence factor in Cm that is essential for the development of wilting symptoms [20, 52]. Hence, the attenuated virulence of the vascular-adapted clones may be a result of their reduced *celA* expression and, consequently, lower cellulase activity. To test this, we investigated whether ectopic expression of *celA* could restore virulence in these vascular-adapted clones. We introduced *celA*, fused to an HA epitope and driven by the *groEL* promoter from *Microbacterium esteraromaticum* (which we previously confirmed to be active in Cm, data not shown), into vascular-adapted clones S2, S4, and S9. CelA accumulation in the transformants was confirmed by restoration of cellulase activity to WT levels in plate assays and by Western blot analysis (Fig. S6A and B).

We inoculated tomato stems with Cm WT, the vascular-adapted clones, or the *celA*-expressing vascular-adapted clones and monitored wilting symptoms. Each CelA-expressing clone displayed increased virulence relative to its parental vascular-adapted clone but remained less virulent than Cm WT (Fig. S6C). This indicates that although restoring cellulase activity partially recovers virulence, it cannot fully compensate for the reduced virulence of the vascular-adapted clones.

### Genomic analyses of vascular- and apoplastic-adapted clones

Vascular and apoplastic adaptation yielded clones with distinct phenotypes that strongly correlated to their adapted tissue. We hypothesized these changes were driven by genomic alterations and fixation of mutations underlying the observed traits. To test this, we sequenced genomes of all representative individual adapted clones used in phenotypic assays after 15 passages from each of the 20 lineages, as well as individual representative adapted clones at passages 5 and 10 to assess mutation fixation over time. Three independent colonies of the parent strain Cm NCPPB382 were also sequenced for comparison. Sequencing was performed on an Illumina Nextseq 2000 platform (150 bp paired-end reads) at the Applied Microbiology Services Laboratory, The Ohio State University. De novo assemblies were generated for all clones and compared with Cm NCPPB382 to detect large deletions and smaller variations via SNP and INDEL calling.

Comparative analysis revealed no large deletions or plasmid loss and mutations were limited to a small set of SNPs and INDELs within the ~3.4 Mb genome of Cm (Table S4). Despite major effects on cellulase activity, no mutations were detected in *celA* or its promoter in vascular-adapted clones. Tracking fixation patterns across passages 5, 10, and 15 showed some mutations arose early and persisted, whereas others appeared only transiently, indicating variable retention of diversity within lineages (Table S4).

Most adapted clones harboured a few mutations in putative coding genes that resulted in gene disruption or shifts in amino acid composition (Tables 1 and S4). Some mutations appeared exclusively in tissue-specific groups. A G308 → A substitution in *CMM_2466*, encoding a putative restriction endonuclease, caused a G103 → D change and was found in four apoplast-adapted clones (L1, L2, L3, L6) but absent in vascular clones (Table 1). Conversely, six vascular-adapted clones carried two independent mutations in *CMM_1284*, absent from apoplast clones: C58 → T in S1, S2, S6 (R20C), and A110 → G in S3, S4, S9 (H37R) (Table 1, Fig. S7A). *CMM_1284* encodes a 107-aa HipB/XRE-type transcriptional regulator, with both substitutions located in its predicted helix-turn-helix DNA-binding domain (Fig. S7B).

**Table 1:** Coding-sequence substitutions and short indels in vascular and apoplast-adapted clones.

Type	Clone	Mutation	Locus tag	Putative annotation	Protein sequence changes
**Vascular adaptation**	S1	C1458114- > T	CMM_1284	Transcriptional regulator, HipB/XRE-type	R20C
	S2	C1458114- > T	CMM_1284	Transcriptional regulator, HipB/XRE-type	R20C
	S3	A1458166- > G	CMM_1284	Transcriptional regulator, HipB/XRE-type	H37R
		C2524625- > G	CMM_2231	Hypothetical protein	L158V
	S4	C1143378- > T	CMM_0996	Two component sensor kinase	A328V
		A1458166- > G	CMM_1284	Transcriptional regulator, HipB/XRE-type	H37R
		A2227810- > G	CMM_1971	Metallo-beta-lactamase	P68S
	S6	C1458114- > T	CMM_1284	Transcriptional regulator, HipB/XRE-type	R20C
	S7	C1187404- > CGCTGCT	CMM_1037	Metal-transporting ATPase	A118- > AAA
	S9	G455804- > T	CMM_0376	Phosphonate ABC transporter, ATPase	L87M
		C561362- > G	CMM_0482	Hypothetical protein	R435G
		C910375- > T	CMM_0806	GntR-family transcriptional regulator	P224S
		A1458166- > G	CMM_1284	Transcriptional regulator, HipB/XRE-type	H37R
	S10	A660294- > G	CMM_0577	O-succinylbenzoate-CoA ligase	E228G
**Apoplastic adaptation**	L1	GCT122241- > G	CMM_0082	Beta-glucosidase	E228- > frame shift
		G848708- > A	CMM_0754	Phosphoribosylamine-glycine ligase	A72V
		C2781803- > T	CMM_2466	Restriction endonuclease	G103D
	L2	C2781803- > T	CMM_2466	Restriction endonuclease	G103D
	L3	C919435- > T	CMM_0813	Acyl-CoA oxidase	A634V
		C2781803- > T	CMM_2466	Restriction endonuclease	G103D
	L6	C2781803- > T	CMM_2466	Restriction endonuclease	G103D
	L7	GGCGACC2690286- > G	CMM_2386	Membrane-bound tyrosin-protein phosphatase	∆A456, ∆T457
	L8	G1758202- > T	CMM_1552	NUDIX hydrolase	A26D
		G2309671- > A	CMM_2046	Hypothetical protein	R20H
	L10	T2397731- > TGCGGCTTC	CMM_2121	Glutathione S-transferase	G196- > frame shift

To determine whether mutations identified in representative clones reflect the broader population within each lineage, we performed amplicon sequencing targeting six coding regions in Cm WT and six cycle 15 populations (CmC15; Tables S5 and S6). These regions were selected based on confirmed SNPs identified in the sequenced representative clones, including variable regions in *CMM_1284* and *CMM_2466*. Although minor variation (<3%) was detected at all loci, indicating limited population heterogeneity, most amplicons contained the SNPs identified in the sequenced representative clones (Fig. S8, Table  S6), indicating strong selection within the populations. These findings reveal specific mutations selected during vascular and apoplastic adaptation, providing a genetic basis for tissue-specific traits.

### CMM_1284 modulates virulence, EPS production, surface attachment, and cellulase activity

**Figure 7:**
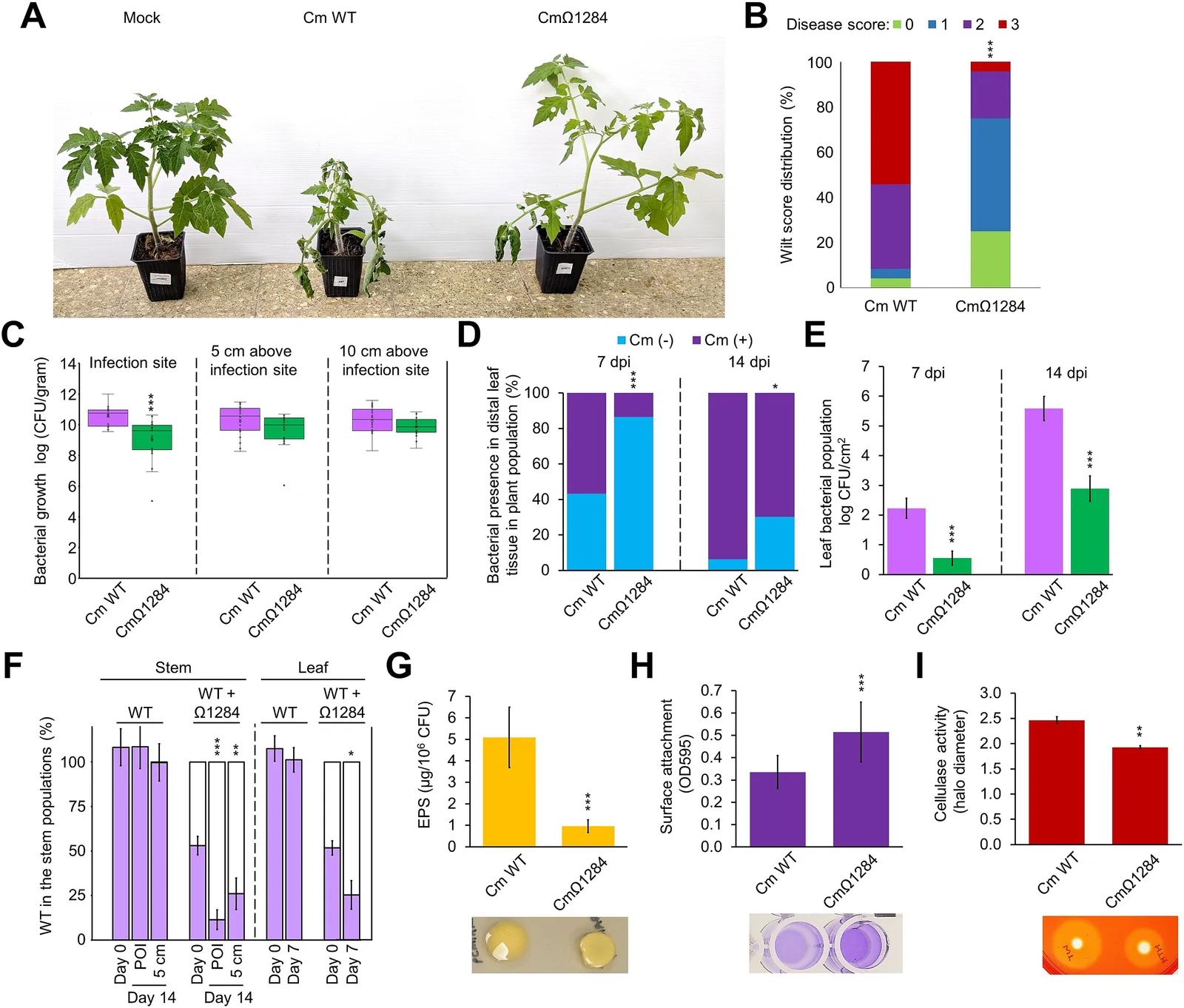
Characterization of the CMM_1284 marker exchange mutant. (A–E) four-leaf stage tomato plants were wound-inoculated with Cm WT and the *CMM_1284* marker exchange mutant (CmΩ1284) at the stem areas between the cotyledons. (A) Representative plants were photographed at 14 days post-inoculation (dpi). (B) Wilt symptoms were scored based on the percentage of wilted leaves using the following scale: 0 = no wilting, 1 = 1%–25%, 2 = 25%–50%, 3 = 51%–100%. The stacked graph shows the distribution of 24 replicates for each clone pooled from three experiments at 14 dpi. (C) Bacterial growth of the indicated clones was monitored in the stem at the point of infection (POI), 5 cm above the POI, and 10 cm above the POI at 7 and 14 dpi. Data are shown in a box plot, representing at least 19 replicates pooled from two experiments. (D, E) bacteria were isolated and quantified from pooled terminal leaflet samples from the second, third, and fourth true leaves of each plant at 7 and 14 dpi. Data represent at least 30 replicates pooled from four (7 dpi) or three (14 dpi) experimental repeats. (D) Stacked Bar graphs represent the presence/absence of bacteria in the sampled leaflets at 7 and 14 dpi. (E) bar graphs show the averages and standard errors of bacterial populations in the leaves at 7 and 14 dpi. (F) Tomato plants were co-inoculated with a 1:1 mixture of WT and CmΩ1284 or WT alone by wound inoculation (vascular) or leaf infiltration (apoplast). Graphs show the percentage of WT within Cm populations in the inoculation solution at time 0 and in stems at the POI and 5 cm above the POI at 14 dpi following vascular inoculation, and in leaf tissues at the infiltration site at 0 and 7 dpi following apoplast inoculation. Graphs represent at least 10 biological repeats pooled from two (apoplast inoculation) or three (vascular inoculation) experiments. (G) Cm WT and CmΩ1284 were spotted (OD600 = 1) on LB agar supplemented with 5% sucrose and incubated for four days. Plates were photographed at 5 dpi (lower panel), and EPS production was quantified in the resulting clones (upper panel) using phenol–sulfuric acid hydrolysis assays followed by colorimetric quantification, standardized to bacterial count. (H) Cm WT and CmΩ1284 were grown on LB medium, standardized to OD600 = 0.5, and incubated in 24-well plates without shaking for 10 days. Supernatants were removed, wells were washed three times with distilled water, and attached bacteria were stained with crystal violet to quantify surface attachment (lower panel), followed by colorimetric quantification (upper panel). (I) Cm WT and CmΩ1284 were spotted (OD600 = 1) onto M9 medium supplemented with 0.1% carboxymethyl cellulose (CMC), and incubated for four days. Plates were stained with 0.1% Congo red, photographed (lower panel), and halo diameters were measured with a ruler to assess CMC degradation (upper panel). (G, H, I) graphs represent means ± SE of at least ten biological replicates pooled from two experiments. (B–I) “^*^” indicate that data were significantly different compared to Cm WT (B, C, D, E, G, H, I) or time 0 (F) (U-test for C, E, G, H, and I; chi-squared test for B and D; paired U-test for F; ^*^P value <.05, ^**^P value <.01, ^***^P value <.001).

Comparative genome analyses identified two independent mutations in the putative transcriptional regulator gene *CMM_1284* across six vascular-adapted clones, all showing attenuated virulence, enhanced surface attachment, and reduced cellulase activity. These findings suggest CMM_1284 contributes to the vascular lifestyle by modulating these traits. To test this, we constructed a *CMM_1284* marker exchange mutant (CmΩ1284; Fig. S7C and D) and examined its impact on traits altered in vascular-adapted clones. CmΩ1284 was first tested for virulence, systemic colonization, stem-to-leaf migration and competitive fitness in tomato. Like most vascular-adapted clones, CmΩ1284-inoculated plants displayed significantly reduced wilting symptoms compared to WT (Fig. 7A and B), whereas systemic colonization in the stem was unaffected (Fig. 7C). In contrast, CmΩ1284 showed reduced stem-to-leaf migration, with lower bacterial frequency and populations in the second to fourth leaves at 7 and 14 dpi (Fig. 7D and E). In plant competition assays with Cm WT, CmΩ1284 populations were overrepresented during both vascular and apoplastic growth (Fig. 7F and S2), indicating that this gene negatively affects Cm competitive fitness *in planta*. Finally, we assessed CmΩ1284 for in vitro virulence-associated traits and found that it formed dry colonies, produced less EPS, exhibited stronger surface attachment, and showed reduced cellulase activity (Fig. 7G–I). These results support CMM_1284 as a regulator of the vascular lifestyle and its associated traits.

### Media-adapted clones do not demonstrate directional phenotypic selection

To confirm that tissue adaptation was due to host selective pressure rather than culture conditions, a control population of ten clones was generated by subculturing on LB agar for 15 passages. These clones (LB1–LB10) were analysed for EPS production, cellulase activity, and virulence. Unlike tissue-adapted clones, which showed consistent shifts, media-adapted clones displayed scattered phenotypes: reduced EPS and cellulase activity in LB1 and LB3, complete cellulase loss in LB8, and altered virulence in LB9 and LB10 (Fig. S9). Thus, prolonged media culturing may cause mutations but not consistent, directional adaptation, likely reflecting clonal drift.

## Discussion

Bacterial pathogens switch strategies within their host to complete their infection cycles, involving changes in behaviour, target tissues, and movement. To adapt to different infection phases, they must overcome diverse challenges and exhibit phenotypic plasticity. We investigated this plasticity by subjecting Cm to tissue-specific experimental evolution, differentiating adaptation to vascular versus apoplastic lifestyles. Ten parallel clones in each condition diverged phenotypically: vascular-adapted clones showed reduced virulence, lower cellulase activity, decreased EPS production, and enhanced surface attachment, whereas apoplast-adapted clones retained virulence, increased EPS production, and reduced surface attachment. These results indicate that xylem and apoplast exert distinct selective pressures, driving directional selection of traits. During disease, Cm exits xylem vessels late in infection, associated with symptom onset. Xylem residence may represent a dormant stage enabling systemic spread without symptoms, whereas apoplastic colonization likely drives vascular collapse and tissue maceration, in a similar manner to previous reports on *E. amylovora* [8]. It is possible that during vascular adaptation in this study, we selected clones that linger longer in the dormant stage, resulting in attenuated virulence. The experimental evolution procedure isolated bacteria at late stages of infection, which may select for bacteria within the stem that tend to persist longer in each tissue. These bacteria may also be exposed to nutrient limitation, which can vary between tissues and potentially influence the selection process. Nevertheless, competition assays indicate that, at least in the case of vascular adaptation, adapted clones exhibit a clear competitive advantage over the parental clone in the main stem. This suggests that, in this specific microenvironment, reduced aggressiveness and limited spread may confer a localized competitive advantage at a particular stage during infection progression, although this may ultimately be detrimental to the bacteria in the long term.

The molecular signals governing the shift from dormancy to virulence are not well defined. In other xylem pathogens such as *E. amylovora* and *Ralstonia* spp., these shifts are linked to population sensing and host/environmental cues like metabolites, physiology, and temperature [53, 54]. Traits consistently observed in tissue-adapted clones suggest strong selection on EPS production and surface attachment, which appear negatively correlated in Cm. In the apoplast, higher EPS may protect against antimicrobial responses [55–57], whereas vascular adaptation favours surface attachment and biofilm formation for xylem colonization [10]. Repeated vascular exposure should thus select for strong attachment and dense biofilms, redundant in the apoplast.

Clones with higher surface attachment produced less EPS. Although EPS usually supports biofilms [50], composition and viscosity often matter more than quantity [51]. For instance, in *X. fastidiosa*, mutants lacking EPS-degrading enzymes overproduce EPS, leading to reduced surface attachment and impaired biofilm formation, similar to what we observed in apoplast-adapted clones [58]. EPS overproduction may inhibit cell-to-cell or surface attachment by masking cell wall-anchored proteins [59]. Previous studies in several Gram-positive Firmicutes, have shown that surface attachment and biofilm formation are mediated by cell wall-bound proteins rather than EPS [59–61]. This suggests that EPS may not contribute significantly to biofilm formation in at least some Gram-positive species, contrasting with widely accepted Gram-negative-based models. This likely underlies attenuated virulence: vascular-adapted clones showed reduced stem-to-leaf migration, possibly due to impaired biofilm disassembly, and decreased vessel blockage, as EPS strongly contributes to wilting. Dynamic EPS modulation thus appears central to Cm colonization: low EPS supports local xylem establishment, whereas increased EPS promotes dispersal and systemic spread. These transitions are probably regulated by physiological and environmental cues.

Another trait in vascular-adapted clones was reduced cellulase and amylase activity. Cellulase, linked to virulence through the secreted glucanase CelA, is essential for symptom development [20, 52]. Cell wall–degrading enzymes can also affect EPS and biofilm disassembly [62, 63], though not for CelA: *celA* mutants lack EPS phenotypes, overexpression does not restore vascular clone EPS traits, and natural isolates show no correlation between cellulase, morphology, or EPS. Therefore, CelA more likely modulates plant cell walls and xylem exit, promoting spread as in *Pectobacterium [**64**]*. Overexpressing *celA* only partially restored virulence in vascular clones, suggesting phenotypes depend more on EPS and surface attachment. Why cellulase was negatively selected remains unclear and warrants further study.

Despite major phenotypic changes, adapted clones accumulated few SNPs/INDELs, with no large deletions or plasmid loss. Mutation rate of ~5 × 10^−10^ per bp per generation align with previous studies [65], though stress may elevate rates via reactive oxygen species [66]. Neither *celA*, its promoter, pCM1, nor EPS-associated clusters were mutated, implying upstream regulatory changes. Indeed, six of ten vascular clones acquired mutations in the transcriptional regulator *CMM_1284*, causing amino acid substitutions in its DNA-binding domain. A marker exchange mutant of *CMM_1284* recapitulated vascular-clone phenotypes: enhanced attachment, reduced EPS and cellulase activity, and attenuated virulence, suggesting a role in vascular–apoplastic shifts. However, some clones lacked *CMM_1284* changes yet showed similar phenotypes, and two vascular clones had no coding mutations at all, suggesting regulatory, structural, or epigenetic mechanisms undetected by Illumina sequencing.

Together, our findings highlight the role of clonal plasticity in the transition between latent and virulent states. They suggest that interactions within the bacterial population help regulate the shift between silent and aggressive infection through changes in EPS production and exoenzyme activity. Although latent behaviour ultimately limits completion of the infection cycle, it can provide a localized fitness advantage in specific microenvironments within defined time frames, underscoring the importance of population-level balance. Our results also refine the role of biofilm in systemic infection. Even though biofilm formation is required for virulence, its establishment appears to correspond to a less aggressive stage, whereas its disassembly and re-establishment, driven by EPS modulation, are key for tuning colonization and disease progression. More broadly, this study illustrates the strength of experimental evolution in uncovering traits associated with localized host interactions in an unbiased manner, revealing functionally relevant phenotypes that may be missed when focusing solely on genetic changes or narrowly defined adaptations.

In summary, experimental evolution identified tissue-specific traits in Cm and revealed that biofilm formation and surface attachment were critical for vascular colonization but limited migration and virulence. This suggests that biofilm disassembly is essential for transitioning from a latent vascular phase to a virulent apoplastic phase.

## Supplementary Material

Supplementary_material_wrag110

## Data Availability

The datasets generated during the current study are available in the NCBI repository, under BioProject PRJNA1333927 (https://www.ncbi.nlm.nih.gov/bioproject/?term=PRJNA1333927).
