# Supplementary material for: Tissue-specific experimental evolution reveals adaptive trade-offs in the plant vascular pathogen Clavibacter michiganensis
Source: ISME J. 2026 May 7;20(1):wrag110. doi: 10.1093/ismejo/wrag110 (PMC13298646; doi:10.1093/ismejo/wrag110)
Supplement: Supplementary_material_wrag110 [file supplementary_material_wrag110.zip › Fig S1.docx]

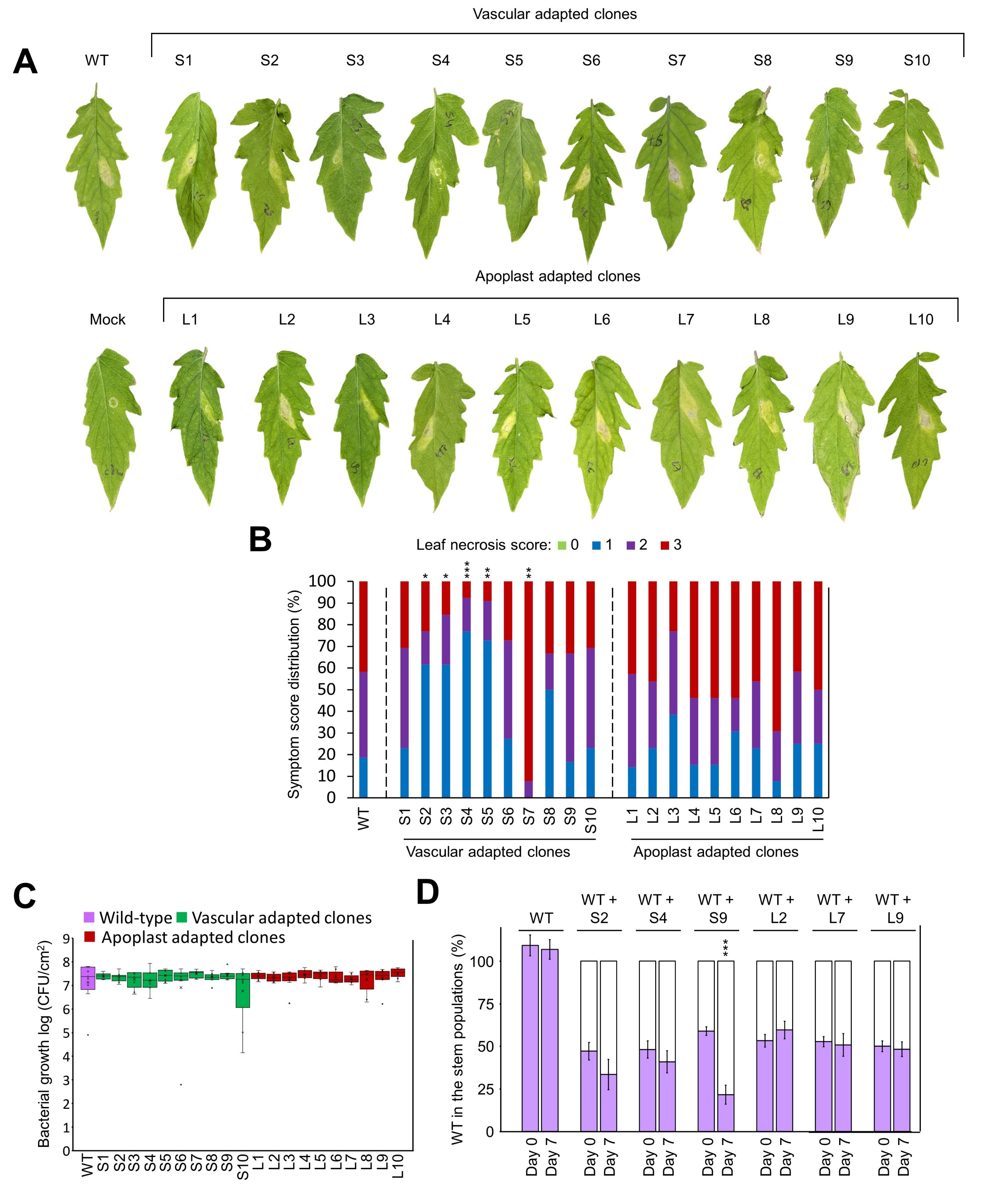


**Fig. S1. Vascular adaptations affect apoplastic virulence. (A-C)** Six-leaf stage tomato leaves were inoculated with the indicated vascular-adapted clones (S1-S10), apoplast-adapted clones (L1-L10) and Cm 382 (WT) through infiltration of bacterial cultures (10^4^ CFU/mL) using a needleless syringe. (**A)** Representative leaves (out of at least 11 repeats) were photographed at 10 days post-infiltration (dpi). **(B)** Necrotic symptoms were scored at 10 dpi according to the following scale: 0 – no symptoms, 1 = chlorosis alone, 2= necrosis of 1-50% of the infected area, 3= necrosis of 51-100% of the infected area. The graph depicts the distribution of at least 11 repeats for each clone pooled from two experiments. "*" represent significant difference (chi-squared test, * *P* value < 0.05, ** *P* value < 0.01, *** *P* value < 0.001) compared to WT. (**C)** Bacterial growth in the infiltration sites at 10 dpi. Data represents 10 repeats for each clone pooled from two experiments. No significant differences (U-test) were observed between each of the clones to the WT. (**D**) Tomato leaves were co-infiltrated with a 1:1 mixture of WT and the indicated adapted clones or WT alone. Graph show the percentage of WT within Cm populations in leaf tissues at the infiltration site at 0 and 7 dpi following apoplast inoculation. Graphs represent 10 biological repeats pooled from two experiments. “*” indicates significant differences compared to day 0 (paired U test, * *P* value < 0.05, ** *P* value < 0.01, *** *P* value < 0.001).
