# Supplementary material for: Tissue-specific experimental evolution reveals adaptive trade-offs in the plant vascular pathogen Clavibacter michiganensis
Source: ISME J. 2026 May 7;20(1):wrag110. doi: 10.1093/ismejo/wrag110 (PMC13298646; doi:10.1093/ismejo/wrag110)
Supplement: Supplementary_material_wrag110 [file supplementary_material_wrag110.zip › Fig S2.docx]

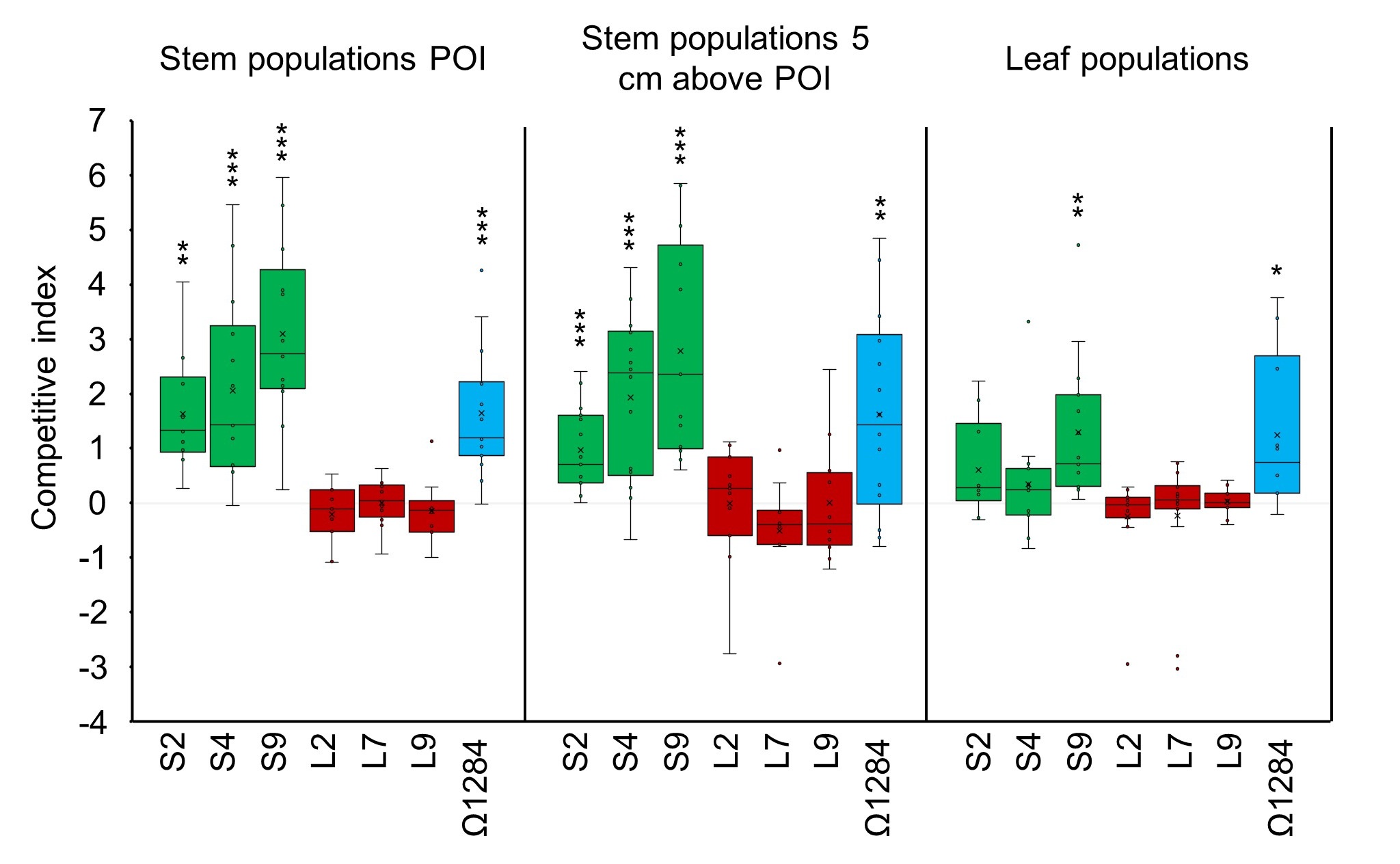


**Fig. S2. Competitive index of tissue-adapted clones.** Indicated clones were mixed 1:1 with Cm WT and inoculated into stems (wound inoculation) or leaves (syringe infiltration). Bacterial populations of Cm WT and the indicated clones were quantified in stems at the point of inoculation (POI) and 5 cm above the POI at 14 days post inoculation (dpi), and in leaves at the site of infiltration at 7 dpi. Box plots show the competitive index (CI), calculated as LOG_10_[(day 0 clone / day 0 WT) / (day 14 or 7 clone / day 14 or 7 WT)], based on at least 10 biological replicates pooled from three (stem) or two (leaf) independent experiments. "*" indicate significant deviation from no competitive difference (CI = 0; Welch’s t-test, * *P* value < 0.05, ** *P* value < 0.01, *** *P* value < 0.001).
