# Supplementary material for: Tissue-specific experimental evolution reveals adaptive trade-offs in the plant vascular pathogen Clavibacter michiganensis
Source: ISME J. 2026 May 7;20(1):wrag110. doi: 10.1093/ismejo/wrag110 (PMC13298646; doi:10.1093/ismejo/wrag110)
Supplement: Supplementary_material_wrag110 [file supplementary_material_wrag110.zip › Fig S3.docx]

**
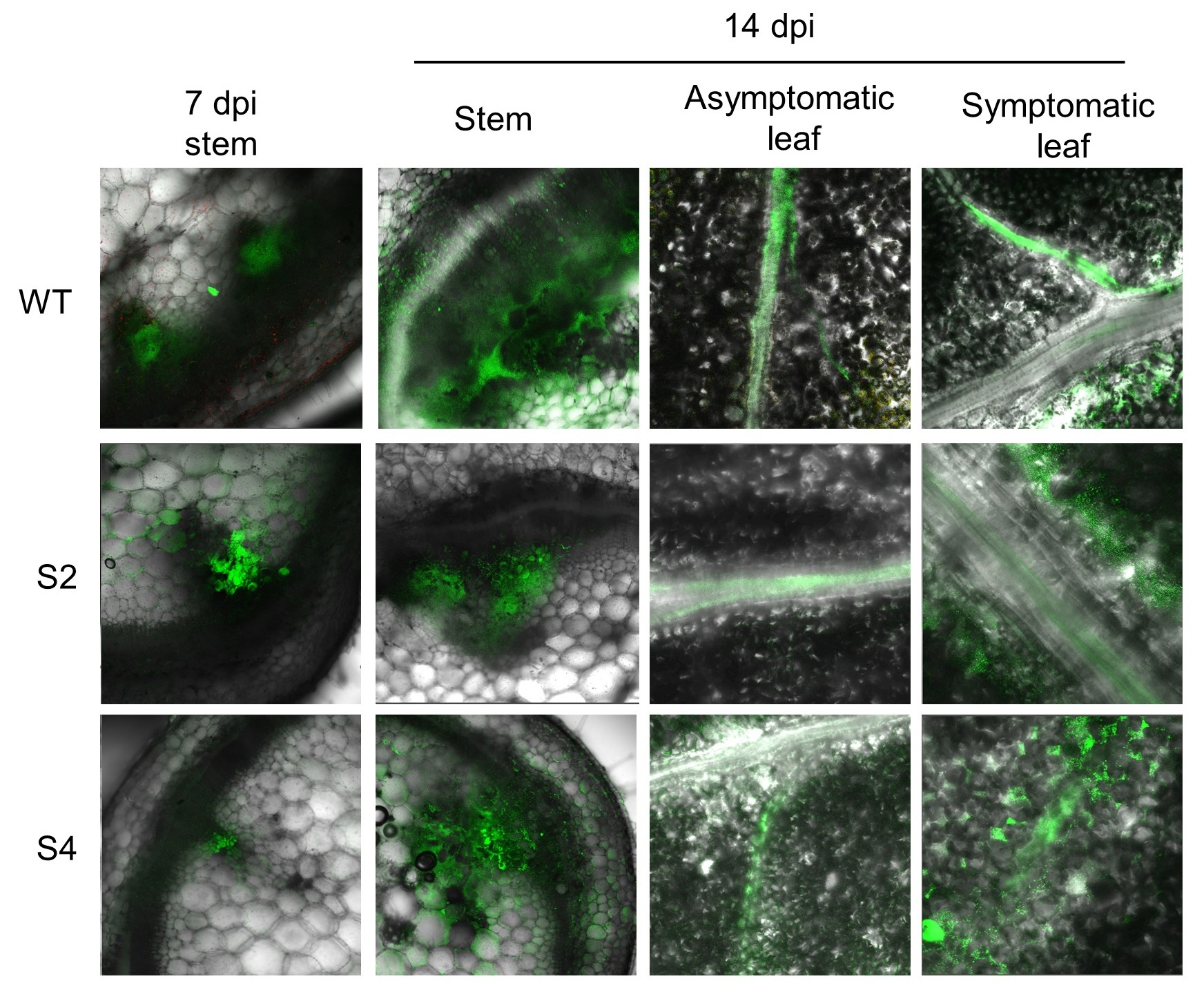
**

**Fig. S3. *In situ* localization of vascular-adapted Cm during infection**. Stem areas between the cotyledons of four-leaf-stage tomato plants were inoculated by wounding with toothpicks soaked in GFP-labeled bacterial suspensions (10^7^ CFU/mL) of Cm WT and the vascular-adapted clones S2 and S4. Bacteria were visualized in horizontal stem cross-sections, as well as in asymptomatic and symptomatic leaves, at 7 and 14 days post-inoculation. The data shown are representative of at least ten biological replicates conducted across three independent experiments.
