# Supplementary material for: Tissue-specific experimental evolution reveals adaptive trade-offs in the plant vascular pathogen Clavibacter michiganensis
Source: ISME J. 2026 May 7;20(1):wrag110. doi: 10.1093/ismejo/wrag110 (PMC13298646; doi:10.1093/ismejo/wrag110)
Supplement: Supplementary_material_wrag110 [file supplementary_material_wrag110.zip › Fig S4.docx]

**
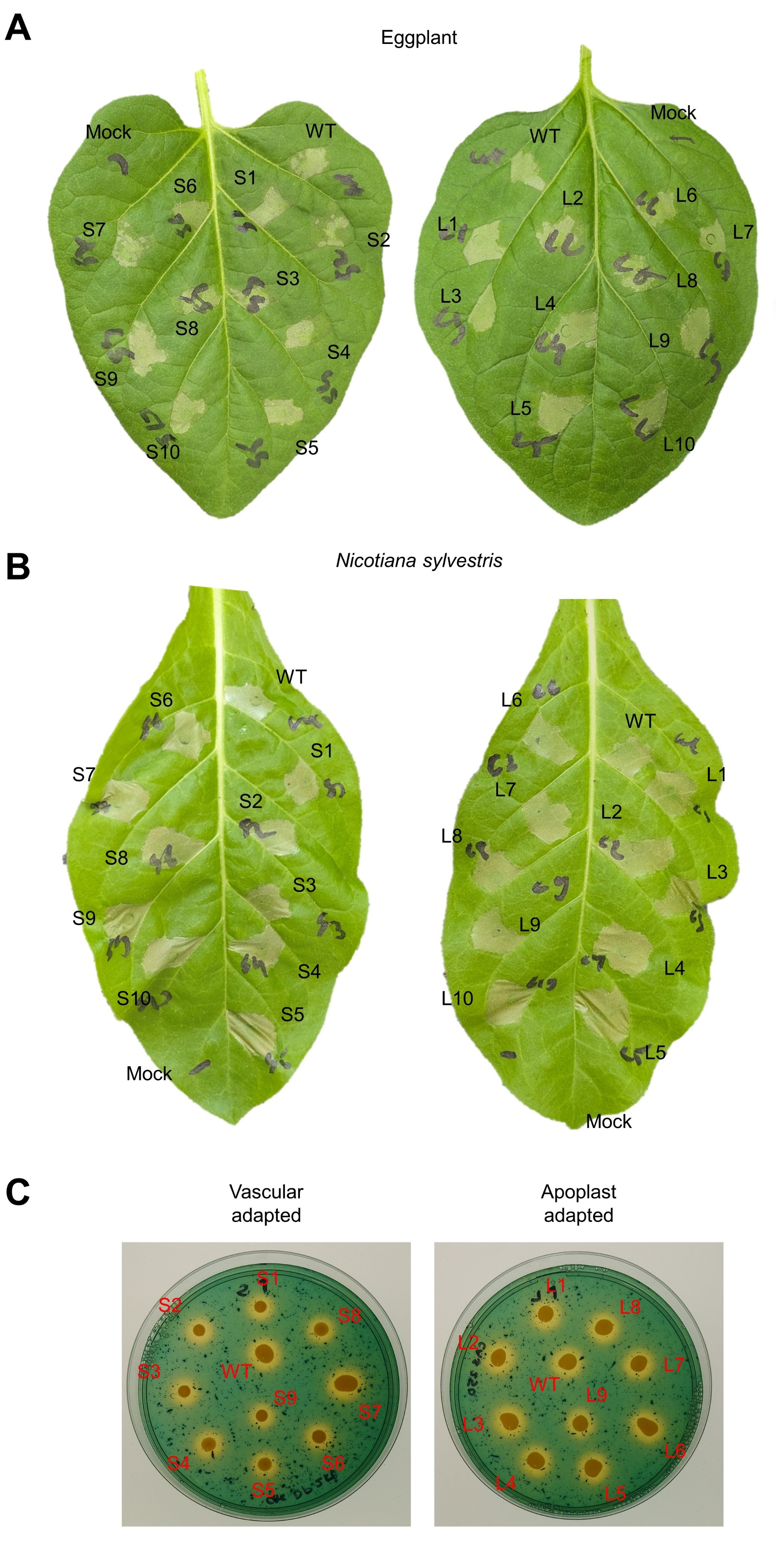
**

**Fig. S4. Vascular and apoplastic adaptations do not affect hypersensitive response induction and siderophore production**. (**A,** **B**) Leaves of six‑leaf‑stage eggplant (**A**) or *Nicotiana sylvestris* (**B**) plants were infiltrated with Cm WT and the indicated vascular- and apoplast-adapted clones using a needleless syringe at a concentration of 5 × 10^7^ CFU/mL. Representative leaves were photographed 36 h post‑infiltration. (**C**) Cm WT and the indicated vascular- and apoplast-adapted clones were spotted (OD600 = 1) on LB agar supplemented with CAS indicator dye and 50 µM 2,2'-dipyridyl and incubated for ten days and photographed. Experiments were repeated twice, with at least five independent replicates per experiment, yielding consistent results.
