# Supplementary material for: Tissue-specific experimental evolution reveals adaptive trade-offs in the plant vascular pathogen Clavibacter michiganensis
Source: ISME J. 2026 May 7;20(1):wrag110. doi: 10.1093/ismejo/wrag110 (PMC13298646; doi:10.1093/ismejo/wrag110)
Supplement: Supplementary_material_wrag110 [file supplementary_material_wrag110.zip › Fig S5.docx]

**
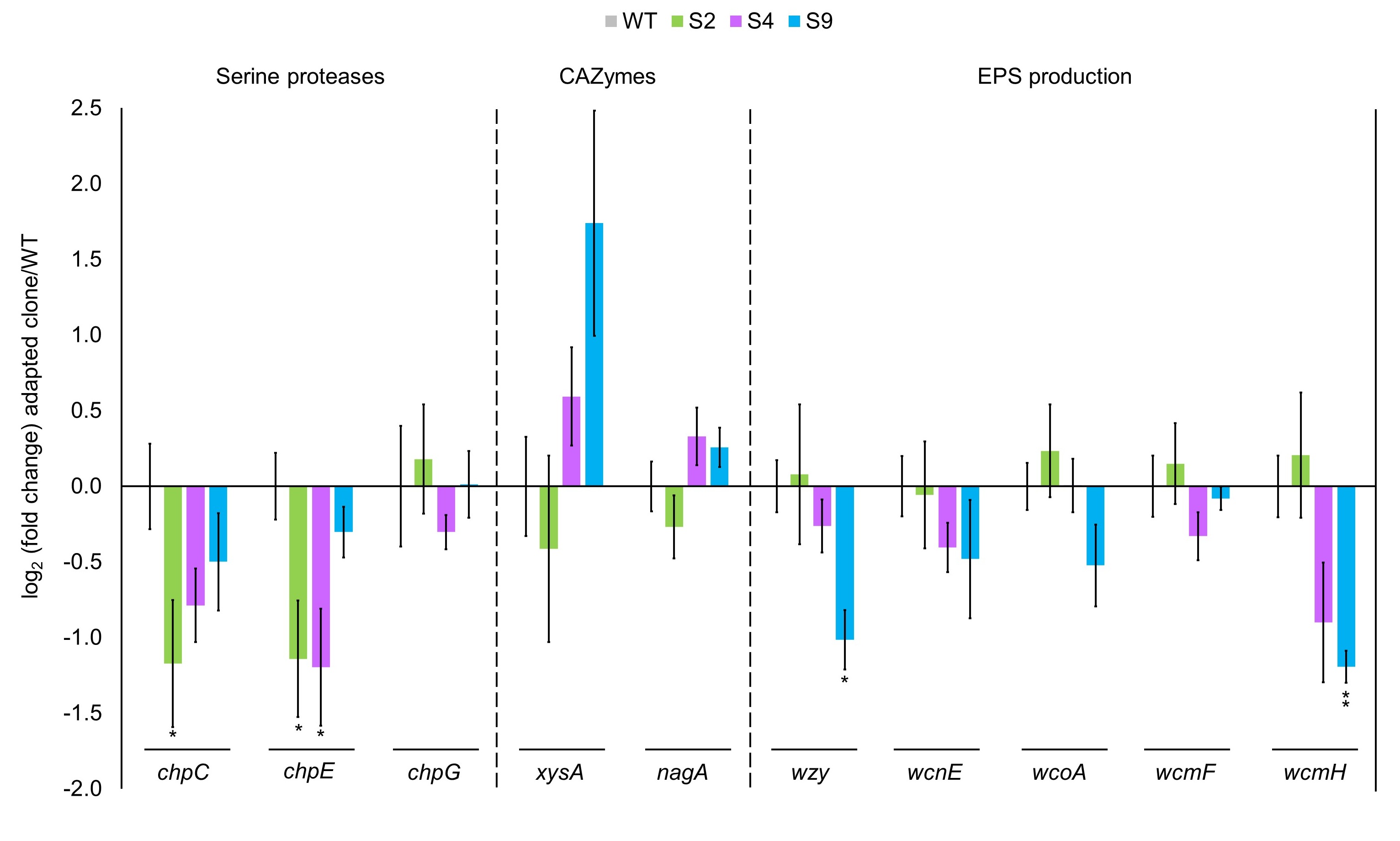
**

**Fig. S5.** **Transcriptional expression of virulence-associated genes in vascular-adapted clones**. mRNA transcript abundance was quantified by RT‑qPCR for the putative serine protease genes chpC (CMM_0052), chpE (CMM_0039), and chpG (CMM_0059), the putative CAZyme genes xysA (CMM_1673) and nagA (CMM_0049), and genes predicted to be associated with EPS production, including wzy (CMM_0715), wcnE (CMM_0718), wcoA (CMM_0819), wcmF (CMM_1597), and wcmH (CMM_1601). Cultures were incubated for 24 h in sucrose‑supplemented M9 medium. gyrA (CMM_0007) was used for normalization. Graph depict the mean ± SE of relative transcript abundance compared to Cm WT, based on six independent biological replicates pooled from two independent experiments. "*" indicate statistically significant differences versus WT (Welch’s t-test, * *P* value < 0.05, ** *P* value < 0.01).
