# Supplementary material for: Tissue-specific experimental evolution reveals adaptive trade-offs in the plant vascular pathogen Clavibacter michiganensis
Source: ISME J. 2026 May 7;20(1):wrag110. doi: 10.1093/ismejo/wrag110 (PMC13298646; doi:10.1093/ismejo/wrag110)
Supplement: Supplementary_material_wrag110 [file supplementary_material_wrag110.zip › Fig S8.docx]

**
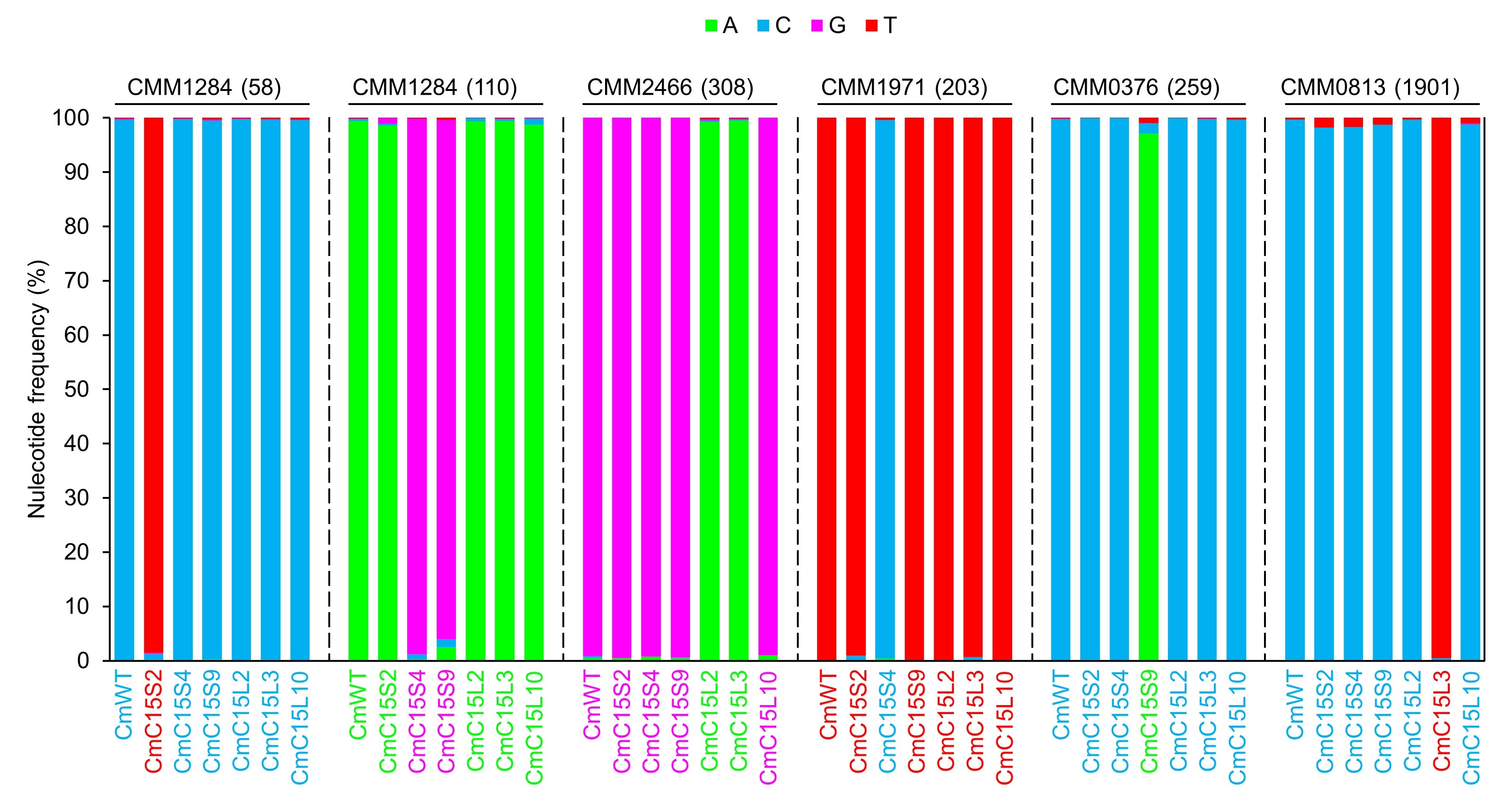
**

**Fig. S8. SNP frequency in adapted clone populations at cycle 15.** Stacked bar graphs show nucleotide frequencies at SNP sites identified in individual adapted clones used for functional assays within clone populations from the indicated lineages at cycle 15 (Table S2). The x-axis is color-coded according to nucleotide identity at each genomic position identified in the sequenced, functionally characterized independent clones.
