# Supplementary material for: Tissue-specific experimental evolution reveals adaptive trade-offs in the plant vascular pathogen Clavibacter michiganensis
Source: ISME J. 2026 May 7;20(1):wrag110. doi: 10.1093/ismejo/wrag110 (PMC13298646; doi:10.1093/ismejo/wrag110)
Supplement: Supplementary_material_wrag110 [file supplementary_material_wrag110.zip › Table S2 new_wrag110.docx]

**Table S2: Plasmids used in this study**

| **Plasmid** | **Relevant characteristic** | **Reference** |
| --- | --- | --- |
| pHN216 | *E. coli-Clavibacter* shuttle vector, based on the replicon of pCM2, Neo^R^/Km^R^, Gnt^R^ | [1] |
| pHN216:p*cel*:*GUS* | pHN216 derivative introduced with *E. coli* *GUS* gene under the control of the Cm *celA* (pCM1_0020*)* promoter. Used for promoter activity assay, Neo^R^/Km^R^ | This study |
| pHN216:p*gyrB*:*GUS* | pHN216 derivative introduced with *E. coli* *GUS* gene under the control of the Cm *gyrB* (CMM_0006*)* promoter. Used for promoter activity assay, Neo^R^/Km^R^ | This study |
| pK2-22 | *E. coli-Clavibacter* shuttle vector, based on the replicon of pCM1, expressing EGFP under the control of the p*CMP1* promoter. Used for in planta localization analyses, Neo^R^/Km^R^ | [2] |
| pMA-RQ:Cmp | Cloning vector for production of Cm integration plasmids carrying the p*CMP1* promoter, MCS and 3×HA tag, Amp^R^ | [3] |
| pCMAT | pMA-RQ:Cmp introduced with ***aac(3)-IV*** upstream to the p*CMP1* promoter, Gnt^R^, Amp^R^ | This study |
| pCMAT:1284 | pCMAT MCS was introduced with a fragment containing the 5' flanking region of CMM_1284-nptII-3' flanking region of CMM_1284. Used for generation of CMM_1284 maker exchange mutant. Neo^R^/Km^R^, Gnt^R^ Amp^R^ | This study |
| pCMIAR | Integrative plasmid for Cm. Cm integration site covering the 89863–90317 region of Cm NPPB382 (AM711867) was introduced downstream to the 3×HA tag of pCMAT. Gnt^R^ Amp^R^ | This study |
| pCMINR | Integrative plasmid for Cm. The ***aac(3)-IV*** resistance gene in pCMIAR was replaced with the *nptII* resistance gene. Neo^R^/Km^R^, Amp^R^ | This study |
| pCMIARG | Modified pCMIAR variant replacing pCMP1 promoter with Microbacterium esteraromaticum groEL (FVO59_01375) promoter (pMe*groEL*). Used for Cm overexpression via genomic integration, Gnt^R^, Amp^R^ | This study |
| pCMIARG:*celA* | pCMIARG MCS introduced with Cm *celA* ORF (pCM1_0020) in frame with the C-terminal 3×HA tag. Used for CelA overexpression. Gnt^R^, Amp^R^ | This study |

*Neo^R^, Km^R^, Gnt^R^ and Amp^R^ indicate neomycin, kanamycin, gentamicin and ampicillin resistance, respectively

**References**

1. Laine MJ, Nakhei H, Dreier J, et al. Stable transformation of the gram-positive phytopathogenic bacterium *Clavibacter michiganensis* subsp. *sepedonicus* with several cloning vectors. Appl Environ Microbiol. 1996;62: 1500–1506. https://doi:10.1128/AEM.62.5.1500-1506.1996

2. Chalupowicz L, Zellermann EM, Fluegel M, et al. Colonization and movement of GFP-labeled *Clavibacter michiganensis* subsp. *michiganensis* during tomato infection. Phytopathology. 2012;102: 23–31. https://doi:10.1094/PHYTO-05-11-0135

3. Verma RK, Roman-Reyna V, Raanan H, et al. Allelic variations in the *chpG* effector gene within *Clavibacter michiganensis* populations determine pathogen host range. PLoS Pathog. 2024;20: e1012380. https://doi:10.1371/journal.ppat.1012380
