# Supplementary material for: Tissue-specific experimental evolution reveals adaptive trade-offs in the plant vascular pathogen Clavibacter michiganensis
Source: ISME J. 2026 May 7;20(1):wrag110. doi: 10.1093/ismejo/wrag110 (PMC13298646; doi:10.1093/ismejo/wrag110)
Supplement: Supplementary_material_wrag110 [file supplementary_material_wrag110.zip › Table S6 new_wrag110.docx]

**Table S6. Nucleotide abundance in representative altered genes in adapted clone populations**

|  | **CMM_1284 (58)^1^** | | | | **CMM_1284 (110)** | | | | **CMM_2466 (308)** | | | | **CMM_1971 (203)** | | | | **CMM_0376 (259)** | | | | **CMM_0813 (1901)** | | | |
| --- | --- | --- | --- | --- | --- | --- | --- | --- | --- | --- | --- | --- | --- | --- | --- | --- | --- | --- | --- | --- | --- | --- | --- | --- |
|  | **A** | **C** | **G** | **T** | **A** | **C** | **G** | **T** | **A** | **C** | **G** | **T** | **A** | **C** | **G** | **T** | **A** | **C** | **G** | **T** | **A** | **C** | **G** | **T** |
| **CmWT** | 2^2^ | 8013 | 14 | 10 | 8004 | 28 | 4 | 13 | 6 | 4 | 1235 | 0 | 0 | 0 | 0 | 29 | 4 | 7995 | 3 | 14 | 1 | 853 | 0 | 3 |
| **CmC15^3^S2** | 9 | 93 | 16 | 7920 | 7936 | 26 | 81 | 7 | 9 | 0 | 1882 | 0 | 0 | 1 | 0 | 109 | 9 | 7992 | 1 | 2 | 0 | 218 | 0 | 4 |
| **CmC15S4** | 5 | 8017 | 6 | 9 | 21 | 73 | 7930 | 13 | 8 | 0 | 1021 | 0 | 1 | 231 | 0 | 1 | 1 | 7994 | 0 | 3 | 0 | 113 | 0 | 2 |
| **CmC15S9** | 15 | 7988 | 19 | 23 | 199 | 120 | 7709 | 31 | 6 | 1 | 1238 | 0 | 0 | 0 | 0 | 62 | 7724 | 148 | 5 | 76 | 0 | 155 | 0 | 2 |
| **CmC15L2** | 5 | 8016 | 7 | 10 | 7996 | 47 | 4 | 3 | 7956 | 19 | 23 | 15 | 0 | 0 | 0 | 64 | 7 | 7987 | 0 | 3 | 0 | 332 | 0 | 1 |
| **CmC15L3** | 12 | 8001 | 10 | 17 | 8007 | 33 | 6 | 10 | 7984 | 15 | 9 | 14 | 0 | 1 | 0 | 154 | 4 | 7980 | 6 | 10 | 2 | 16 | 1 | 3674 |
| **CmC15L10** | 1 | 7995 | 9 | 24 | 7938 | 88 | 7 | 7 | 9 | 0 | 876 | 0 | 0 | 0 | 0 | 113 | 7 | 7977 | 2 | 23 | 0 | 406 | 1 | 4 |

^1^Brackets indicate the represented nucleotide position within the ORF

^2^Number of reads corresponding to the indicated nucleotide within clone population

^3^C15 represents pooled clone population isolated from the indicated lineage at cycle 15 which was used as template for amplicon sequencing.
